# Magnitude and variability of blood pressure and renal vascular conductance responses to postural changes, exercise, and cold in black adults: A pilot study

**DOI:** 10.14814/phy2.70888

**Published:** 2026-05-05

**Authors:** Mohamed H. Ibrahim, Alison J. McLure, Mary J. Jackson, Brittany Harrison, Sarah Brockley, Elizabeth C. Jones, A. Parker Ruhl, Hans C. Ackerman

**Affiliations:** ^1^ Physiology Unit, Laboratory of Malaria and Vector Research National Institute of Allergy and Infectious Diseases Rockville Maryland USA; ^2^ Department of Radiology and Imaging Sciences NIH Clinical Center Bethesda Maryland USA; ^3^ Critical Care Medicine and Pulmonary Branch National Heart Lung and Blood Institute Bethesda Maryland USA

**Keywords:** autonomic nervous system, blood pressure, renal circulation, ultrasonography, Doppler, vasoconstriction

## Abstract

We developed a noninvasive approach to measure vascular responses to sympathetic stimuli. We hypothesized that standing, handgrip exercise, or cold exposure would raise blood pressure (BP) and lower renal vascular conductance (RVC). BP was recorded continuously and renal arterial velocity was measured using renal Doppler ultrasound. Repeated measures one‐way ANOVA with Dunnett's post hoc test was used to compare BP during stimulation against baseline. Intraclass correlation analysis was used to compare first and second visits. Seven women and four men aged 29.9 ± 4.7 years participated. On first visits, standing from supine raised systolic BP by 11.9 ± 2.6 mmHg (*p* = 0.0018) and diastolic BP by 15.0 ± 1.9 mmHg (*p* < 0.0001). Handgrip exercise raised systolic BP by 15.9 ± 4.1 mmHg (*p* = 0.0146) and decreased systolic RVC by 0.68 ± 0.26 cm/s/mmHg (*p* = 0.0049). Cold increased systolic BP by 27.8 ± 3.4 mmHg (*p* < 0.0001) and decreased systolic RVC by 0.32 ± 0.07 cm/s/mmHg (*p* = 0.0049). BP and RVC responses were similar from visit one to visit two; however, most outcome measurements were not correlated within individuals. These findings expand our understanding of vasoregulation in Black adults and guide studies to characterize vascular function in the future.

## 1 INTRODUCTION

Acute vasoconstrictive responses are driven primarily by the sympathetic nervous system (Katayama & Saito, 2019). Sympathoexcitatory stimuli such as orthostatic stress, isometric handgrip exercise, and cold exposure elicit changes in blood pressure (BP) through rapid vasoconstriction of resistance arteries (Drew et al., 2017; Teixeira & Vianna, 2022). These physiological stimuli can be used to probe the range of normal vascular responses in humans, where prior work has demonstrated that these autonomic stress tests elicit changes in muscle sympathetic nerve activity, blood pressure, and renal blood flow (Carter, 2019; Chapman et al., 2019; Drew et al., 2017; Maier et al., 2025; Momen et al., 2003; Teixeira et al., 2023). Renal vascular conductance (RVC) responses to these stimuli remain relatively understudied, due in part to the technical challenges of collecting blood pressure and renal arterial velocity data simultaneously in humans. RVC is an important indicator of the state of vasoconstriction of small arteries distal to the renal artery (Díaz‐Morales et al., 2023; Joyce et al., 2019; Lerman & Rodriguez‐Porcel, 2001) and may reflect an individual's vascular health more broadly (Geraci et al., 2025).

We hypothesized that RVC would decrease in response to vasoconstrictive stimuli. To address this hypothesis, we measured BP and renal blood flow responses to three different stimuli: standing, isometric handgrip exercise, and cold exposure. To assess RVC, we obtained continuous blood pressure readings simultaneously with renal artery Doppler ultrasound measurements of blood flow velocity.

The goal of this pilot study was to develop and test a standardized, noninvasive, and cardiac phase–specific approach to assess renal vascular conductance during autonomic stress. This framework provides a method for quantifying renal vasoconstrictive responses that can be applied in future studies aimed at characterizing determinants of human vascular reactivity, such as the roles of iron, hemoglobin, and NO signaling in feedback vasodilation (Brooks et al., 2025; Dora et al., 1997, 2000; Dunaway et al., 2024).

## 2 METHODS

### 2.1 Inclusion and exclusion criteria

Inclusion criteria for enrollment on the clinical protocol 19‐I‐0093 “Collection of Human Biospecimens for Basic and Clinical Research Into Globin Variants” included the ability to provide informed consent, willingness to provide biological samples, and agreement to allow genetic testing and storage of biospecimens for future use. Exclusion criteria for enrollment on this protocol included pregnancy, positive testing for hepatitis B virus, hepatitis C virus, or HIV, or any medical condition requiring immediate intervention.

A history and physical exam were performed, including review of medications and health history, specifically whether the subject has a history of diabetes, hypertension, or hypercholesterolemia, history of repeated episodes of syncope, or Raynaud's phenomenon (cold or numbness in fingers in response to cold).

Blood samples were collected for hemoglobin electrophoresis, glucose‐6‐phosphate dehydrogenase (G6PD) deficiency, and alpha thalassemia genetic testing. We tested for HIV, Hepatitis B and C, and nicotine metabolites. A basic metabolic panel, lipid panel, and hemoglobin‐A1C testing were performed. Standardized measurements of height and weight were performed to determine body mass index (BMI).

For the analyses presented here, additional exclusions were applied using a pre‐specified, documented, standard operating procedure to ensure the participants were eligible. Individuals were excluded from this analysis if they self‐reported coronary artery disease, angina, heart failure, or prior myocardial infarction, or if they had any of the following hematologic conditions: sickle cell trait, beta thalassemia trait, alpha thalassemia silent carrier or trait, or G6PD deficiency. Individuals were excluded if they were currently taking medication to treat hypertension, diabetes mellitus, or hypercholesterolemia, or reported repeated episodes of syncope or a history of Raynaud's phenomenon. Individuals were excluded from this analysis if they had any of the following: age greater than or equal to 40 years, BMI greater than or equal to 35.0 kg/m^2^, systolic blood pressure (SBP) greater than or equal to 139 mmHg, diastolic blood pressure (DBP) greater than or equal to 89 mmHg, hemoglobin A1c greater than or equal to 7.0%, or serum cotinine level greater than 10 ng/mL (signifying exposure to nicotine). Individuals self‐reporting use of nicotine‐containing products or marijuana within 30 days of noninvasive vascular measurements were also excluded from this analysis.

### 2.2 Noninvasive vascular measurement visit

Each subject participated in two visits, in which the same study procedure was conducted at least 48 h apart. The second visit was included to assess inter‐day variability and reproducibility of BP and renovascular responses to vasoconstrictive stimuli under standardized conditions. Before each visit, participants were instructed to fast 12 h prior to the study visit and drink approximately 16 oz. of water in the 12‐h period prior to the study visit. Participants were instructed to avoid caffeine, nonsteroidal anti‐inflammatory drugs, decongestant medications, dietary supplements, and phosphodiesterase inhibitors 12 h prior to the study visit; if these instructions were not followed, participants were rescheduled or excluded. Each study visit was conducted between the hours of 7 am and 11 am to limit the effects of diurnal variation. The clinic room temperature goal range was 22°C–26°C, and the achieved temperature was 23.8°C ± 0.8°C. Each visit began with a review of interim health history, medications, and adherence to study instructions as detailed in the standard operating procedure (Data S1). To evaluate participant hydration status, a urine sample was collected and urine specific gravity was measured using a refractometer (Optika, Italy, SN642842). To proceed with the study, participants had to have a urine specific gravity less than 1.035 signifying that they were not dehydrated (Popowski et al., 2001).

### 2.3 Blood pressure brachial cuff measurements

Baseline blood pressure was measured with a brachial cuff at the beginning of the study using a protocol adapted from Rakotz et al. (2017). Participants were seated in a chair with their back and arms supported and with legs uncrossed for 15 min. Participants were asked not to speak or use their cell phone. BP measurements were performed in triplicate using an appropriate‐sized cuff and an automated sphygmomanometer (Omron 907XL) that was calibrated every 6 months.

### 2.4 Continuous finger cuff noninvasive blood pressure measurements

Continuous finger cuff BP measurements were performed using a Human Non‐Invasive Blood Pressure (NIBP) Nano device connected to a PowerLab data acquisition unit (model number PL3508, AD Instruments). Data recording and event marking were performed using LabChart software (Version 8.1.29, AD Instruments). The NIBP device was placed on the participant's non‐dominant hand and two finger cuffs were placed on the middle and pointer fingers. The height correction unit (HCU) was taped to the participant's chest just to the right of the sternum between the third and fourth rib, which approximates the height of the right atrium. The HCU was calibrated prior to each study session. Participants were instructed to refrain from speaking and to lie supine with feet uncrossed and hands at their sides during the study session. Participants underwent a 5‐min rest period in the supine position prior to the start of data collection. A Biometrics LTD isometric hand grip dynamometer (model number G200, AD Instruments) was used to measure, display, and record hand grip force data in LabChart.

### 2.5 Renal ultrasound

Renal ultrasound was performed by a Registered Vascular Technologist (RVT) or a Registered Diagnostic Medical Sonographer (RDMS) using a General Electric Healthcare Logiq S8 ultrasound unit with a convex array ultrasound transducer probe (C1‐6VN—1.0 MHz—6.0 MHz). Static multiplanar anatomic images of the right kidney were obtained at the first visit only. Vascular measurements were recorded on a standardized case report form and were reviewed by a lead vascular ultrasound technician for consistency and accuracy of measurements. Ultrasound images were uploaded to Philips Vue PACS (Philips; Amsterdam, The Netherlands) and reviewed by an attending radiologist. Any incidental findings were reported.

Renal vascular Doppler imaging was performed using an anterior subcostal acoustic window with the transducer oriented in a transverse plane to visualize the proximal right renal artery while the participant lay supine (Data S2). Doppler measurements were obtained from the proximal main renal artery to ensure a stable insonation angle and minimize motion artifacts. This location was selected to provide consistent and reproducible measurements across timepoints. Prior studies have demonstrated that Doppler indices from the main renal artery reflect overall renal hemodynamics and correlate with intrarenal measurements (Ikee et al., 2005; Radermacher et al., 2001). Electrocardiogram monitoring (GE Healthcare; Chicago, IL U.S.) was utilized to assist with interpretation of the Doppler waveforms. Renal Doppler ultrasound images were captured over pre‐specified intervals during isometric handgrip exercise and at baseline, during the final 5 s of the cold pressor test (CPT), and at 5 and 10 min post exposure. Velocity measurements were taken at peak systolic velocity (PSV) and at end diastolic velocity (EDV).

### 2.6 Adrenergic stimuli

Three adrenergic stimuli were administered to elicit a vasoconstrictive response: an orthostatic maneuver, isometric handgrip exercise, and CPT.

#### 2.6.1 Orthostatic maneuver

For the orthostatic maneuver, baseline finger cuff BP recordings were taken in the supine position before patients were instructed to stand. Standing BP measurements were taken after 1 min and 3 min in the standing position in accordance with standardized clinical procedures from the Centers of Disease Control and Prevention (2017). The transition from supine to standing introduces anatomic shifts that disrupt the acoustic window needed for accurate and continuous insonation of the renal artery; therefore, renal Doppler images were not obtained during the orthostatic maneuver.

#### 2.6.2 Isometric handgrip exercise

Isometric handgrip exercise was performed in a supine position with the dominant arm horizontal at their side while holding the handgrip dynamometer with fingers at a 90° angle. Each hand grip effort was sustained for 15 s, and continuous finger cuff SBP and DBP along with renal Doppler ultrasound measurements at PSV and EDV were analyzed over the final 5 s of the 15 s exertion. Three baseline measurements of finger cuff SBP, DBP, and renal doppler velocities (PSV and EDV) were taken at rest, after which the participant was asked to squeeze at their maximum effort for 15 s. This was repeated three times, with a 1‐min rest period after each effort. The average force over all three maximum efforts was calculated. Each participant was asked to squeeze at 30%, 50%, 70%, and 100% of their highest average maximum force, using a real‐time visual guide of the effort target.

#### 2.6.3 Cold pressor test

For the CPT, a 15‐L bucket of cold water was prepared. Ice was removed prior to hand insertion. The goal was to achieve a temperature of 3°C–5°C and the mean (SD) measured temperature was 3.8°C ± 0.6°C across all participants. After baseline measurements, the participant's dominant hand was submerged up to the ulnar process of the wrist for 1 min. Continuous finger cuff NIBP readings were recorded during the 1‐min cold exposure and BP and Doppler images taken in the final 5 s of the exposure were analyzed. This window was selected to capture the peak phase of the cold pressor response, during which sympathetic and blood pressure responses progressively increase during cold exposure (Victor et al., 1987) while allowing stable acquisition of renal Doppler signals. The participant remained in the supine position for 10 min after the cold pressor stimulus was completed and continuous NIBP finger cuff readings with simultaneous renal ultrasound measurements were performed 5 and 10 min after cold exposure.

### 2.7 Data extraction

Events were marked in LabChart during data acquisition and were subsequently extracted from the saved files. For each stimulus, height‐corrected SBP, DBP, and MAP were calculated. Renal Doppler measurements were labeled to correspond with events in LabChart to facilitate analysis of simultaneously recorded blood pressure and renal artery velocity measurements.

### 2.8 Statistical methods

Data were tested for normality using the D'Agostino and Pearson test, and QQ plots were inspected for each outcome measure. Values not normally distributed were log transformed, and a D'Agostino and Pearson test for lognormality was performed. RVC was calculated in systole as the PSV divided by SBP and in diastole as the EDV divided by DBP. Pressure, velocity, and conductance measurements were plotted as mean with standard error. Repeated measures one‐way ANOVA with Dunnett's post hoc test was used to compare physiological responses during each stimulus to corresponding baseline values. This repeated measures approach allows each individual to be compared against their own baselines for testing of statistical significance while allowing us to display non‐normalized data to accurately convey the variation between individuals for each outcome measure. For the graded isometric handgrip exercise, repeated‐measures one‐way ANOVA test for linear trend was performed to assess trends between effort levels (30%, 50%, 70%, and 100% of MVE) and blood pressure, renal arterial velocities, and renal vascular conductance. To assess test–retest reliability between Visit 1 and Visit 2, intraclass correlation coefficients (ICCs) were calculated using a two‐way mixed‐effects model described as ICC (Katayama & Saito, 2019; Teixeira & Vianna, 2022) by Koo and Li (2016).

## 3 RESULTS

Thirteen research participants underwent noninvasive vascular testing consisting of BP and renal arterial Doppler ultrasound measurements in response to orthostatic maneuver, hand grip exercise, and cold exposure. Two subjects were excluded from the analysis due to incomplete data. All participants self‐identified their race as Black or African American and met the criteria for participation as documented by the inclusion/exclusion criteria and the characteristics presented in Table 1. The magnitude and variation of responses to each stimulus during first visits are reported below, along with test–retest reproducibility. Percent changes are provided in the Tables S1–S4, and data from second visits are provided in the figures and Tables S2 and S4.

**TABLE 1:** Demographic and clinical laboratory characteristics of the study participants.

Participants, *N*	11
Age, years	29.9 ± 4.7
Sex	
Male, *N* (%)	4 (26)
Female, *N* (%)	7 (64)
Race	
Black or African American, *N* (%)	11 (100)
Ethnicity	
Non‐Hispanic or Latino, *N* (%)	9 (82)
Hispanic or Latino, *N* (%)	2 (18)
Height, cm	169.7 ± 9.4
Weight, kg	79.7 ± 18.7
BMI, kg/m^2^	27.2 ± 4.6
Systolic blood pressure, mmHg	112.5 ± 10.7
Diastolic blood pressure, mmHg	70.4 ± 5.6
Hemoglobin, g/dL	14.1 ± 1.6
Hemoglobin A1C, %	5.2 ± 0.4
Total cholesterol, mg/dL	182.6 ± 28.9
Creatinine, mg/dL	0.87 ± 0.1
Blood urea nitrogen, mg/dL	14.1 ± 7.4
Urine specific gravity, g/mL	1.016 ± 0.012

*Note*: Values are mean ± standard deviation except where otherwise indicated. SBP and DBP were recorded on initial history and physical visit from the nondominant arm.

Abbreviation: cm, centimeter; g/dL, grams per deciliter; kg, kilogram; kg/m^2^, kilograms per square meter; *n*, number.

### 3.1 Systolic and diastolic blood pressures increase in response to standing, isometric handgrip exercise, and cold exposure

#### 3.1.1 Orthostatic maneuver

**FIGURE 1:**
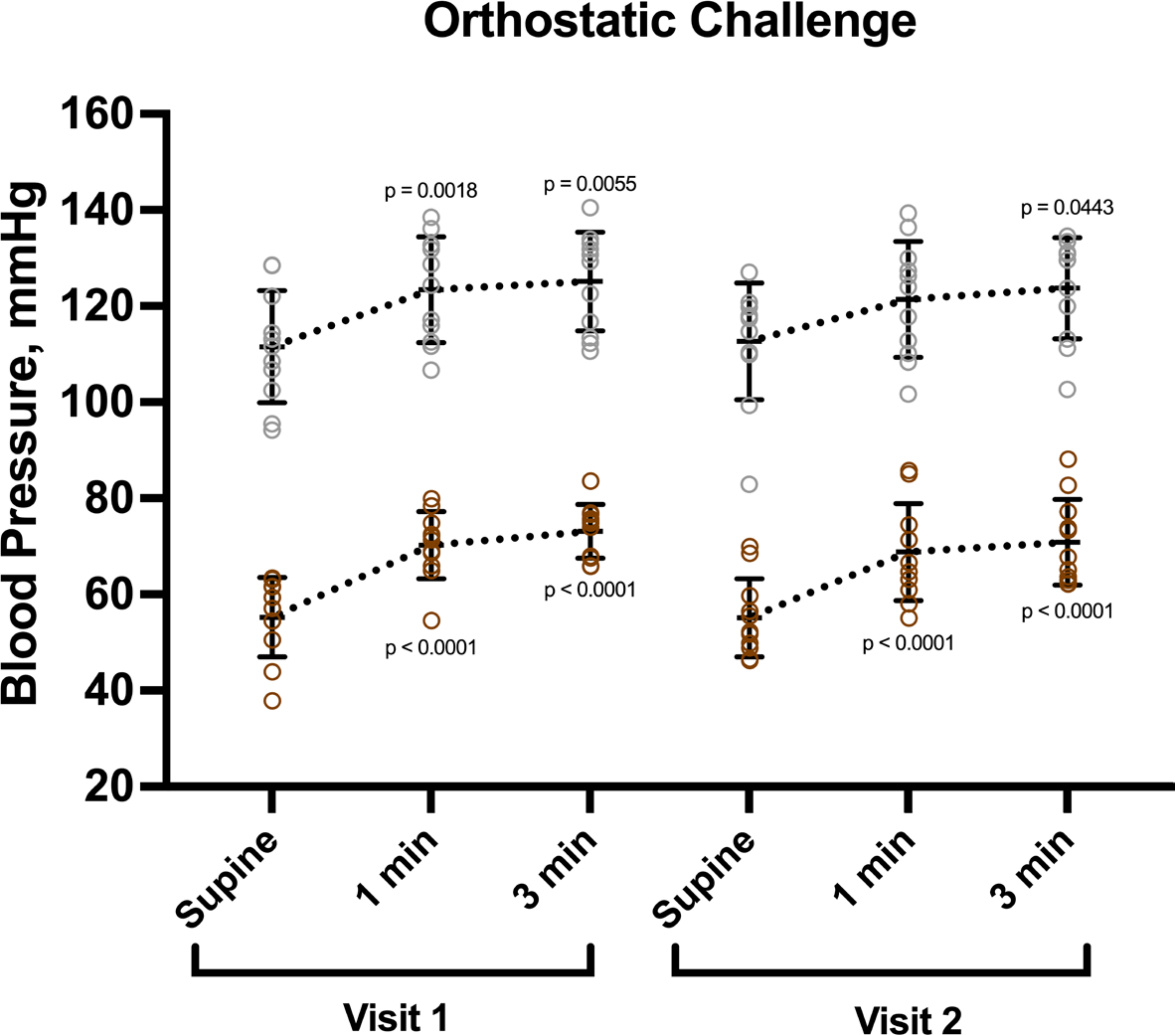
Systolic and diastolic blood pressures increase in response to postural changes. Blood pressure was measured while supine and 1 and 3 min after standing. Data points represent mean blood pressure and error bars represent standard deviation. Upper data points represent systolic blood pressure and lower data points diastolic blood pressure. Statistical significance was assessed using one‐way ANOVA with Dunnett's test, comparing each timepoint to baseline. mmHg, millimeters of mercury.

To assess the vascular response to the adrenergic stimulus of standing from a supine position, BP was measured while the participant was lying supine and at one and 3 min after standing. SBP was 111.5 ± 11.7 mmHg while supine and rose to 123.4 ± 11.0 mmHg 1 min and 125.0 ± 10.3 mmHg 3 min after standing, increases of 11.9 ± 8.5 mmHg (*p* = 0.0018) and 13.6 ± 11.5 mmHg (*p* = 0.0055), respectively (Figure 1 and Table S1). DBP was 55.3 ± 8.3 mmHg while supine and rose to 70.3 ± 7.0 mmHg 1 min and 73.2 ± 5.6 mmHg 3 min after standing, increases of 15.0 ± 6.4 mmHg (*p* < 0.0001) and 17.9 ± 7.0 mmHg (*p* < 0.0001), respectively. SBP responses 1 min after standing were not correlated between visits (intraclass correlation coefficient ICC = 0.3338; *p* = 0.1578; Figure S1). Similarly, there was no correlation of DBP responses to standing between visits (ICC = 0.0; *p* = 0.5).

#### 3.1.2 Isometric handgrip exercise

**FIGURE 2:**
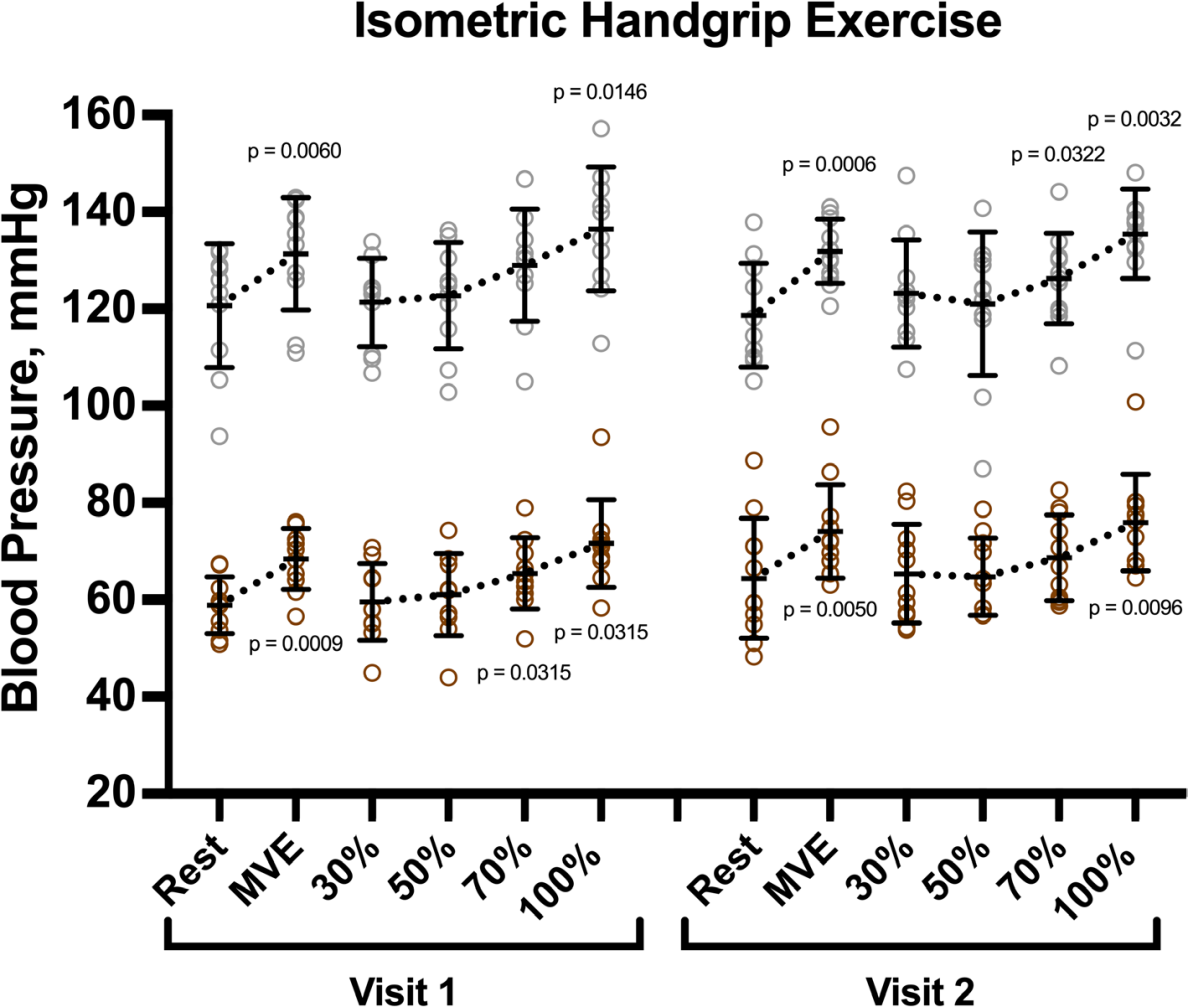
Systolic and diastolic blood pressures increase in response to isometric handgrip exercise. Blood pressure was measured at rest, at maximum voluntary effort (MVE), and at 30%, 50%, 70%, and 100% of MVE. Data points represent mean blood pressure and error bars represent standard deviation. Upper data points represent systolic blood pressure and lower data points diastolic blood pressure. Statistical significance was assessed using one‐way ANOVA with Dunnett's test, comparing each time point to baseline. mmHg, millimeters of mercury.

To assess cardiovascular responses to isometric handgrip exercise, BP was measured at rest, at maximum voluntary effort (MVE), and then at progressive efforts of 30%, 50%, 70%, and 100% of MVE. SBP was 120.5 ± 12.7 mmHg at baseline and increased to 131.2 ± 11.6 mmHg at MVE, a change of 10.7 ± 8.9 mmHg (*p* = 0.0060) (Figure 2 and Table S1). DBP increased from 58.6 ± 5.9 mmHg at baseline to 68.1 ± 6.3 mmHg at MVE, a change of 9.5 ± 9.9 mmHg (*p* = 0.0009). Both SBP and DBP increased as effort levels rose (SBP: *r* ^2^ = 0.8587, *p* = <0.0001; DBP: *r* ^2^ = 0.8745 *p* = <0.0001; Figure S4). Both SBP and DBP responses to exercise displayed moderate correlation between visits (SBP ICC = 0.6509, *p* = 0.0150; DBP ICC = 0.5440; *p* = 0.0418; Figure S1).

#### 3.1.3 Cold pressor test

**FIGURE 3:**
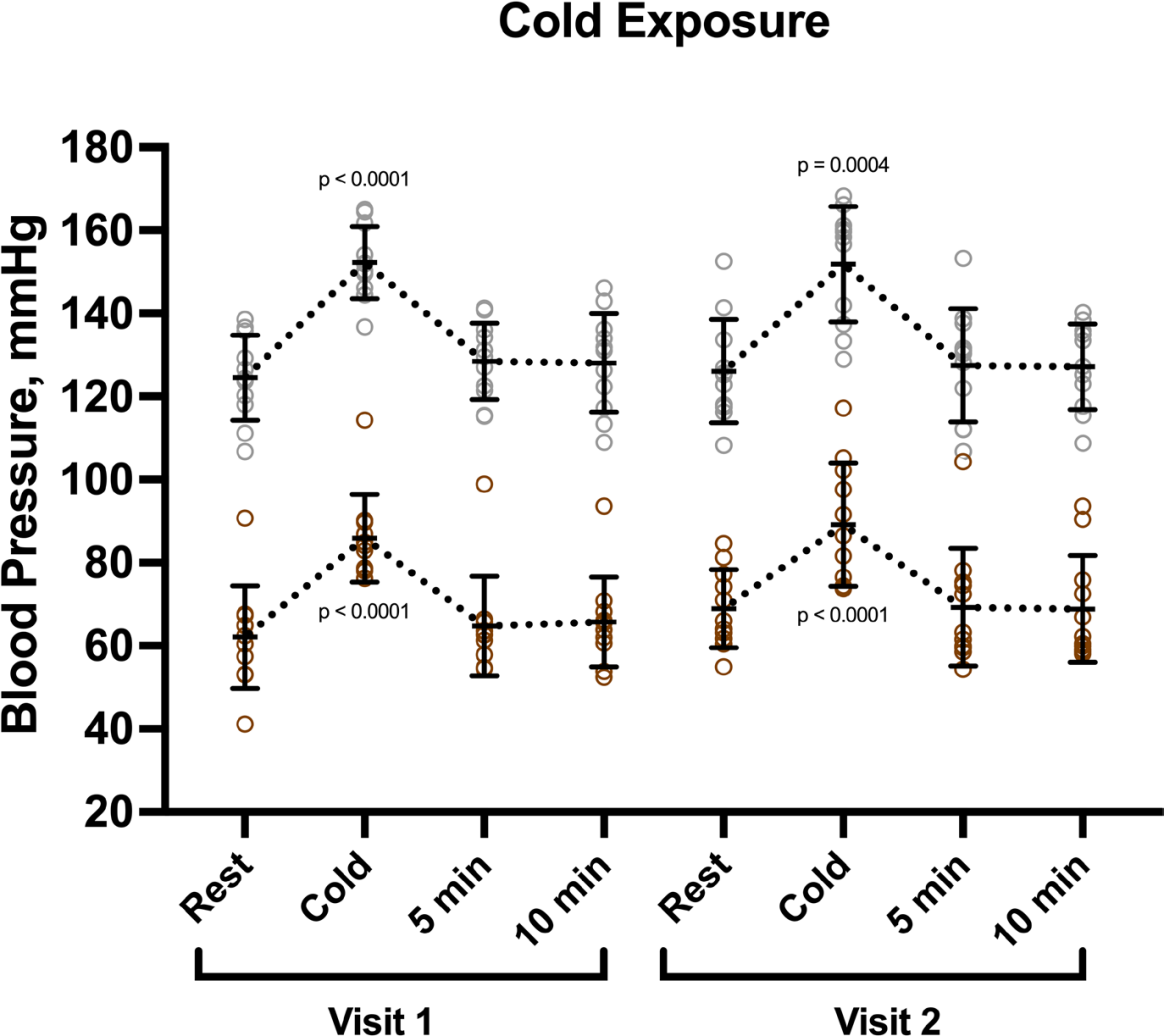
Systolic and diastolic blood pressures increase in response to cold exposure. Cold exposure was performed by immersing the dominant hand in cold water for 1 min. Systolic and diastolic blood pressures were measured on the nondominant arm at rest, after 1 min of cold exposure, and 5 and 10 min after cold exposure ended. Data points represent mean blood pressure and error bars represent standard deviation. Upper data points represent systolic blood pressure and lower data points diastolic blood pressure. Statistical significance was assessed using one‐way ANOVA with Dunnett's test, comparing each time point to baseline. mmHg, millimeters of mercury.

To assess cardiovascular responses to CPT, BP was measured at rest, at the end of a 1‐min immersion of the dominant hand in cold water, and at 5 and 10 min after the hand was removed from cold water. SBP was 124.6 ± 10.2 mmHg at rest and rose to 152.4 ± 8.7 mmHg after 1 min of cold exposure, a maximal increase of 27.8 ± 11.4 mmHg (*p* < 0.0001) (Figure 3 and Table S1). SBP then fell to 128.6 ± 9.2 mmHg 5 min and 128.2 ± 11.8 mmHg 10 min after cold exposure ended. DBP was 62.1 ± 12.3 mmHg at rest and increased to 85.8 ± 10.6 mmHg after 1 min of cold exposure, a maximal increase of 23.8 ± 8.1 mmHg (*p* < 0.0001). DBP then fell to 64.7 ± 12.0 mmHg 5 min and 65.69 ± 10.8 mmHg 10 min after cold exposure ended. SBP and DBP responses to cold exposure were not correlated between visits (SBP ICC = 0.0153; *p* = 0.4812, DBP ICC = 0.0849; *p* = 0.4020; Figure S1).

### 3.2 Peak systolic and end diastolic renal artery velocities decrease in response to isometric handgrip exercise or exposure to cold

#### 3.2.1 Isometric handgrip exercise

**FIGURE 4:**
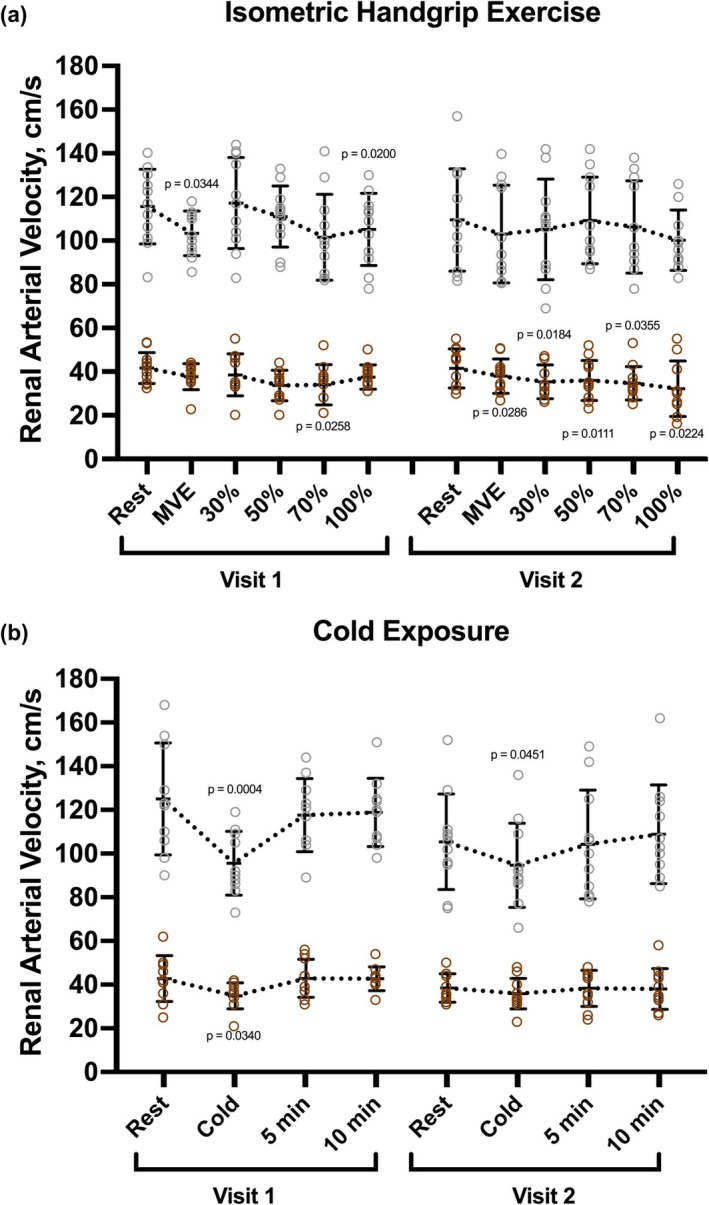
Renal arterial peak systolic and end diastolic velocities decrease in response to isometric handgrip exercise or cold exposure. (a) Isometric handgrip exercise was performed and peak systolic and end diastolic renal arterial blood flow velocities were measured at rest, at maximum voluntary contraction (MVE), and at 30%, 50%, 70%, and 100% of MVE. (b) Cold exposure was performed by immersing the dominant hand in cold water for 1 min. Peak systolic and end diastolic renal arterial blood flow velocities were measured at rest, after 1 min of cold exposure, and 5 and 10 min after cold exposure ended. Data points represent mean renal arterial blood flow velocities and error bars represent standard deviation. Statistical significance was assessed using one‐way ANOVA with Dunnett's test, comparing each time point to baseline. cm/s, centimeters per second.

To assess renal arterial blood flow responses to isometric handgrip exercise, renal artery peak systolic velocity (PSV) and end diastolic velocity (EDV) were measured during isometric handgrip exercise. PSV declined from 115.6 ± 17.1 cm/s at rest to 103.3 ± 10.2 cm/s at MVE, a change of 12.3 ± 12.6 cm/s (*p* = 0.0344) (Figure 4 and Table S1). EDV decreased from 41.7 ± 7.1 cm/s at rest to 37.8 ± 6.0 cm/s at MVE, a change of 3.9 ± 6.3 cm/s (*p* = 0.2232). While a significant decrease in EDV was not observed, there was a decreasing trend in both EDV and PSV with increasing effort (PSV: *r* ^2^ = 0.7477, *p* = 0.0013; EDV: *r* ^2^ = 0.3643, *p* = 0.0319; Figure S5). PSV responses to MVE between visits one and two were moderately, but not significantly, correlated (ICC = 0.5154; *p* = 0.0524; Figure S2). EDV responses to MVE were not correlated between visits (ICC = 0.0; *p* = 0.5).

#### 3.2.2 Cold pressor test

To assess renal arterial blood flow responses to cold exposure, renal artery PSV and EDV were measured at rest, after 1 min of cold exposure, and 5 and 10 min after exposure ended. PSV decreased from 125.0 ± 25.6 cm/s at baseline to 95.6 ± 14.6 cm/s after 1 min of cold exposure, a decrease of 29.4 ± 12.6 cm/s (*p* = 0.0004) (Figure 4 and Table S1). EDV was 42.8 ± 10.5 cm/s at baseline and decreased to 34.9 ± 6.0 cm/s after 1 min of cold exposure, a decrease of 7.9 ± 8.5 cm/s (*p* = 0.0340). PSV and EDV responses to cold between visits one and two were not correlated (PSV ICC = 0.0, *p* = 0.5; EDV ICC = 0.0843, *p* = 0.4086; Figure S2).

### 3.3 Renal arterial vascular conductance decreases during isometric handgrip exercise or exposure to cold

#### 3.3.1 Isometric handgrip exercise

**FIGURE 5:**
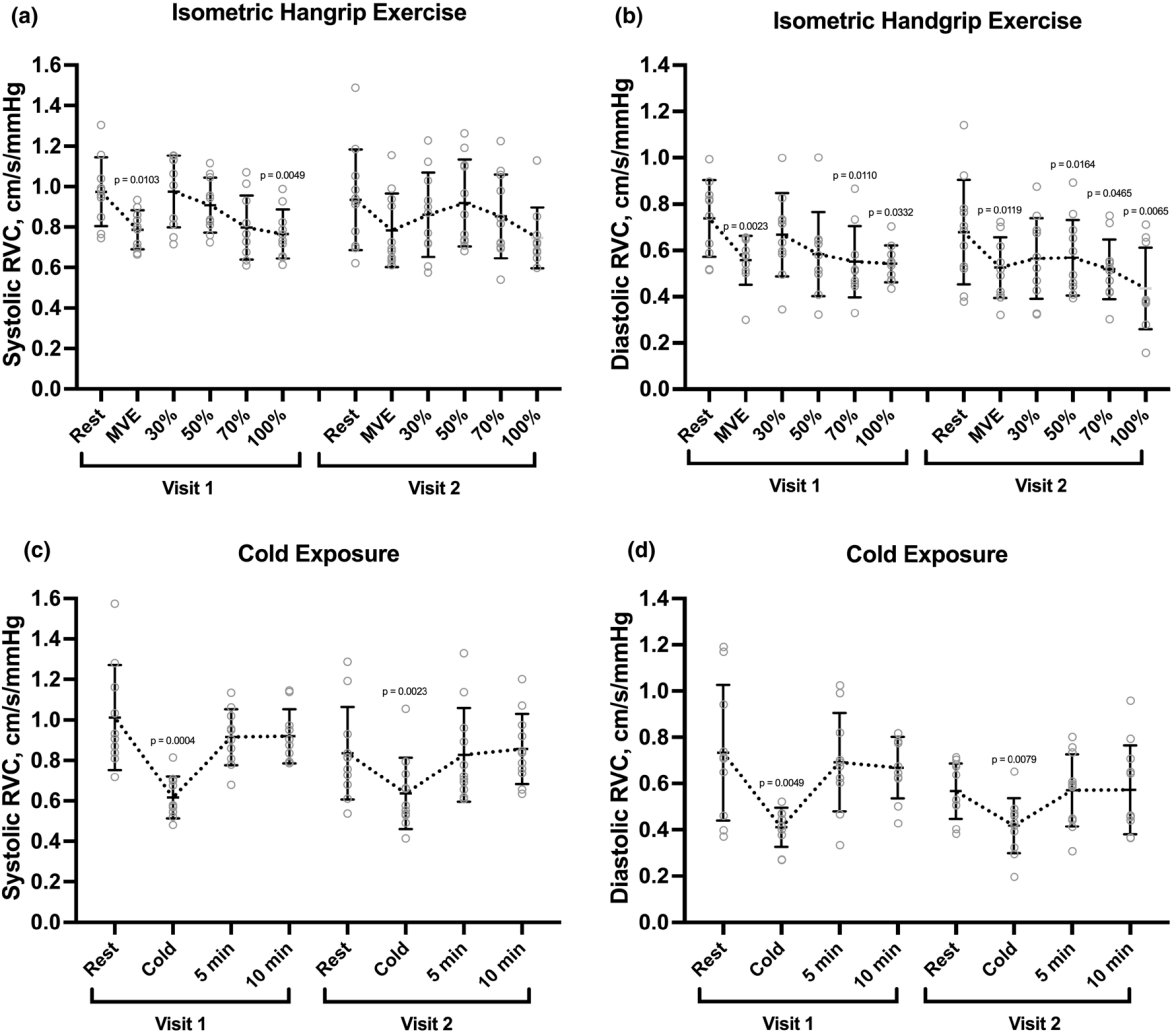
Renal vascular conductance (RVC) decreases in response to isometric handgrip exercise or cold exposure. Renal vascular conductance was calculated by dividing peak systolic velocity (PSV) or end diastolic velocity (EDV) by systolic or diastolic blood pressure, respectively. (a) RVC during systole in response to isometric handgrip exercise at rest, maximum voluntary contraction (MVE), and 30%, 50%, 70%, and 100% of MVE. (b) RVC during systole in response to cold exposure: At baseline, after 1 min of cold exposure, and at 5 and 10 min post‐exposure. (c) RVC during diastole in response to isometric handgrip exercise at rest, MVE, and 30%, 50%, 70%, and 100% of MVE. (d) RVC during diastole in response to cold exposure at baseline, after 1 min of cold exposure, and at 5 and 10 min post‐exposure. Data points represent mean RVC and error bars represent standard deviation. Statistical significance was assessed using one‐way ANOVA with Dunnett's test, comparing each time point to baseline. cm/s/mmHg, centimeters per second per millimeter of mercury.

To assess the renovascular responses to hand grip exercise, renal vascular conductance (RVC) was calculated by dividing the PSV or EDV by the synchronously measured SBP or DBP, respectively. Systolic RVC declined from 0.9740 ± 0.16 cm/s/mmHg at rest to 0.7866 ± 0.10 cm/s/mmHg at MVE, a change of 0.19 ± 0.14 cm/s/mmHg (−19%; *p* = 0.0103) (Figure 5 and Tables S1 and S3). Diastolic RVC decreased from 0.7374 ± 0.17 cm/s/mmHg at rest to 0.5565 ± 0.11 cm/s/mmHg at MVE; a change of 0.18 ± 0.12 cm/s/mmHg (−25%; *p* = 0.0023). Additionally, both systolic and diastolic RVC decreased with increasing exercise effort (Systolic RVC: *r* ^2^ = 0.9172, *p* = <0.0001; Diastolic RVC: *r* ^2^ = 0.9146, *p* = 0.0002; Figure S6). The systolic RVC responses to exercise between visits one and two were moderately correlated (ICC = 0.6414; *p* = 0.0167; Figure S3) while the diastolic RVC responses were not correlated (ICC = 0.1843; *p* = 0.2937).

#### 3.3.2 Cold pressor test

To assess the renovascular responses to CPT, RVC was measured at rest, following a 1‐min cold exposure, and at 5 and 10 min after the cold exposure ended. Systolic RVC decreased from 1.012 ± 0.26 cm/s/mmHg at rest to 0.6166 ± 0.10 cm/s/mmHg after 1 min of cold exposure, a decrease of 0.32 ± 0.14 cm/s/mmHg (−39%; *p* = 0.0049; Figure 5 and Tables S1 and S3). Diastolic RVC was 0.7338 ± 0.29 cm/s/mmHg at baseline and dropped to 0.4110 ± 0.08 cm/s/mmHg after cold exposure, a decrease of 0.40 ± 0.13 cm/s/mmHg (−44%; *p* = 0.0004), the largest change for any combination of stimulus and outcome measure. Systolic and diastolic conductance responses after exposure to cold were not correlated between visits (Systolic RVC ICC = 0.0, *p* = 0.5; Diastolic RVC ICC = 0.0, *p* = 0.5; Figure S3).

## 4 DISCUSSION

This study provides a framework for quantifying and understanding renal vasoconstrictive responses to adrenergic stimuli while providing important reference data on renal and systemic vascular responses in Black adults. To understand the systemic and renal hemodynamic effects of vasoconstrictive adrenergic stimuli in humans, we obtained continuous BP readings with synchronous renal arterial velocities at rest and in response to postural changes, isometric handgrip exercise, and CPT. The simultaneous BP and velocity measures allowed us to measure RVC to assess the function of small arteries within the human kidney during autonomic stress. Each stimulus elicited an increase in BP and a decrease in renal arterial blood flow velocity consistent with prior reports of renal vasoconstriction during adrenergic stress in healthy humans (Drew et al., 2017; Katayama & Saito, 2019; Momen et al., 2003). Subsequently, RVC decreased in response to isometric handgrip exercise and cold exposure. The observation that renal arterial blood flow velocity decreased despite increases in systemic blood pressure implies that renal resistance arteries, distal to the renal artery, constricted in response to these stimuli. The observed response validates this noninvasive approach as a physiologically sensitive method for assessing renal arterial function during autonomic stress, supporting its use in future studies of human renovascular function.

The cardiovascular response to standing involves gravitational redistribution of blood volume into the venous system of the lower extremities, reducing cardiac filling and stroke volume. This results in a fall in blood pressure that activates carotid baroreceptors which trigger vasoconstriction of resistance arteries to support systemic BP. Participants in our study raised their SBP and DBP after standing from a supine position (Ringer et al., 2025). This differs from prior descriptions of the response to standing, in which SBP may briefly decrease and DBP increase slightly within the first seconds after postural change before compensatory increases occur (Ringer et al., 2025). Another study showed that in healthy adults, SBP is higher in the supine position compared to sitting or standing (Eşer et al., 2007). There are several ways in which our study differed from these prior reports of orthostatic BP changes. First, we ensured participants were not hypovolemic at the time of study by prescribing fluid intake in the 12 h before the study and by testing urine specific gravity prior to the study session to exclude dehydration, a known cause of orthostatic hypotension. Second, standard brachial cuff pressure measurements may be affected by changes in the vertical distance between the brachial cuff and the heart upon standing. In our study, a continuous height correction sensor was used to mitigate the effects of postural changes on the nominal BP reading. Interestingly, the observation that increases in both SBP and DBP following the transition to standing in euvolemic adults, suggests that compensatory mechanisms act rapidly, to not only correct the initial drop in pressure but to overshoot, resulting in higher BP readings after standing.

Two primary reflex pathways contribute to the cardiovascular response to exercise: the mechanoreflex and the metaboreflex. The mechanoreflex, mediated by group III afferents, responds rapidly and is the primary driver of the immediate sympathetic pressor responses elicited during brief muscle contractions (Kaufman & Hayes, 2002; Momen et al., 2003). In contrast, the metaboreflex typically requires 30 s or longer for sufficient metabolite accumulation to activate group IV afferents (Boushel, 2010; Katayama & Saito, 2019). Prior human studies support this temporal distinction, demonstrating that static muscle contraction or passive stretch rapidly evokes renal vasoconstriction via mechanoreflex pathways (Drew et al., 2017; Momen et al., 2003). Consistent with these findings, we observed decreases in renal artery blood flow velocity and RVC during brief isometric handgrip exercise. Because each muscle contraction in our study lasted 15 s, mechanoreflex‐mediated sympathetic activation was the primary stimulus increasing SBP and DBP and decreasing RVC. We observed a progressive rise in BP with increasing effort, suggesting the mechanoreflex signal is proportional to effort. Doppler ultrasound revealed decreases in renal artery blood flow velocity in response to exercise, consistent with a mechanism in which renal arteries distal to the main renal artery constrict to reduce renal blood flow (Rocha et al., 2023).

Sympathetic activation during cold stress is intensity dependent and influenced by the populations of thermal and pain receptors that are stimulated (Greaney et al., 2016). The cold pressor response, elicited here by submerging one hand in cold water for 1 min, is primarily a nociceptive response and is unlikely to involve thermoregulatory mechanisms which require more time for body temperature to fall (Burton et al., 2016; Castellani & Young, 2016). This nociceptive response increases both cardiac output and systemic vascular resistance to raise BP (Delong & Sharma, 2025; Freemas et al., 2024). In our participants, 1 min of cold exposure raised SBP and DBP, displaying strong and rapid systemic responses consistent with prior studies examining cardiovascular responses to cold exposure (Chapman et al., 2019; Teixeira et al., 2023). Renal artery blood flow velocity decreased PSV and EDV, despite the higher systemic BP perfusing the renal artery. A prior study of patients with vasospastic disorders found that when patients immersed their hand into 4°C ice water, radial arterial blood flow dropped significantly (Karabacak et al., 2015). Our study, which likewise utilized 4°C water for the CPT, demonstrated similar decreases in blood flow in the renal artery.

In this study, RVC was calculated during systole and diastole using synchronized finger cuff plethysmography and renal Doppler ultrasonography of the proximal right renal artery. Renal arterial blood flow velocity decreased despite concurrent increases in systemic blood pressure, revealing significant reductions in RVC during both systole and diastole in response to adrenergic stimuli. RVC decreases in response to cold exposure were consistent with prior evidence that sympathetic adrenergic fibers mediate vasoconstriction during cold exposure (Karabacak et al., 2015; Victor et al., 1987). Several studies have combined blood pressure and renal arterial velocity measurements to assess renal vascular dynamics during sympathetic activation. Momen et al. (2003) and Drew et al. (2017) reported increases in renal vascular resistance during handgrip exercise or passive calf stretch, while Teixeira et al. (2023) demonstrated reductions in RVC during both isometric handgrip and cold exposure, reflecting organ‐specific sympathetic regulation of the renal vasculature. These studies relied on MAP‐based or single‐point blood pressure normalization, which lacks continuous alignment with the cardiac cycle. By contrast, synchronizing beat‐to‐beat blood pressure with renal arterial velocities in this study enabled resolution of RVC within discrete cardiac phases, allowing for phase‐specific reductions to be interpreted with greater temporal specificity. Confirming and extending prior observations, we observed substantial RVC reductions during maximal isometric handgrip exercise or cold exposure, indicating rapid renal vasoconstriction across both phases of the cardiac cycle.

To evaluate day‐to‐day variability and reproducibility of BP and renal vascular responses to adrenergic stimuli, participants completed a second visit using the same standardized operating procedure. While most BP and renal vascular responses to postural changes, exercise, and cold did not differ between visits at the group level, many of the responses were not significantly correlated between visits. An exception to this general finding was observed for SBP, DBP, and systolic RVC responses during the isometric handgrip exercise, where each response showed a moderate correlation between visit one and visit two. The day‐to‐day variability in other vascular responses may reflect differences in sympathetic nervous system activation following initial exposure versus a repeated exposure to the same stimuli, where a degree of acclimatization or comfort with the study procedure may have diminished the strength of these stimuli at second visits. Such differences could result in diminished magnitudes of physiological responses at second visits, even though the direction of change remained the same. For example, the change in renal artery PSV after cold exposure differed between visits one and two. Catecholamine sensitivity may also have played a role. α₁‐adrenergic receptors, which mediate vascular smooth muscle contraction, play a critical role in the vasoconstriction response used to assess RVC (Rudner et al., 1999). There is evidence that α‐adrenergic receptors can desensitize with repeated stimulation, which may partly explain the variation in responses between first and second visits (Kiuchi et al., 1992). Further research in humans is needed to better understand the effects of repeated exposures to sympathetic stimuli and the extent and variability of receptor desensitization.

This study has both strengths and limitations. We sought to limit impacts of diet and hydration status by instructing participants to fast for 12 h prior to the study visit and to drink approximately 16 oz. of water during that time. We measured urine specific gravity on the study day to assess hydration status and to exclude participants if they were dehydrated. Participants were further instructed to avoid caffeine, NSAIDs, decongestants, dietary supplements, and phosphodiesterase inhibitors during the 12 h preceding the study session. However, individual differences in caffeine and alcohol consumption or exercise greater than 12 h prior to testing could have potentially influenced autonomic and hemodynamic responses on the morning of the study. Study visits were conducted at the same time each day (7–11 am) to control for diurnal effects and room temperature was controlled to limit thermal effects on vascular regulation (Cui et al., 2007). Great care was taken to accurately determine resting BP in participants by performing protocolized brachial cuff BP readings on each arm in triplicate to ensure physiologically normal BP before testing. All subjects received the same scripted instructions and coaching throughout the study, including prompts to maintain steady breathing during handgrip to minimize inadvertent Valsalva maneuvers.

Nevertheless, inter‐day variability in the magnitude of vascular responses to adrenergic stimuli within individuals was observed. These differences could be due to unmeasured participant‐level factors such as psychological stress, mood, or unreported changes in health that together represent normal day‐to‐day variation within an individual. Attenuated blood pressure and sympathetic nerve activity responses due to biological sex may also influence autonomic and vascular responses through sex hormones, differences in muscle mass, and variations in sympathetic transduction efficiency (Hart et al., 2009; Minson et al., 2000). Normalizing handgrip effort to each participant's maximum voluntary effort and analyzing all hemodynamic and renovascular responses relative to individual baselines helped mitigate sex‐related variability, but potential hormonal or sex‐specific differences such as menstrual cycle phase, which was not monitored, may still have contributed to inter‐day variation (Lee & Millar, 2024; Minson et al., 2000; Tharpe et al., 2023). Differences in technique between ultrasound technicians could have played a role; however, all technicians were trained by one lead technician who performed quality assurance of renovascular images and measurements. Doppler measurements were performed using a standardized protocol and documented on structured case‐report forms, and all ultrasonographers were either a Registered Vascular Technologist (RVT) or a Registered Diagnostic Medical Sonographer (RDMS). Due to technical and anatomic limitations of abdominal ultrasound, it was not feasible to measure renal artery velocity in standing participants because transition from supine to standing prevents maintenance of a stable insonation window. Therefore, we were unable to assess the renal responses to postural changes. Inherent inter‐day variability in the performance of devices used for measurement could have also contributed to differences in measured vascular responses between different individuals or between visits of the same individual. Although beat‐to‐beat BP and renal blood flow velocity data were collected continuously, analyses were performed using predefined time windows rather than the full time course of responses, which may limit characterization of the temporal dynamics of these responses. We acknowledge that the cohort size (*n* = 11) is modest; however, as a pilot study employing a repeated‐measures design, it provided sufficient resolution to detect physiologically meaningful outcomes across stimuli. The absence of a comparison group from other racial or ethnic backgrounds limits the ability to directly compare the magnitude of these responses with those reported in prior studies of predominantly non‐Black cohorts. Physiological and technical sources of variability limited the reproducibility of some vascular measures, but understanding this variability informs the design of future studies that may benefit from larger sex‐balanced cohorts, enhanced technical standardization, and continued efforts to ensure consistent conditions across visits.

## 5 CONCLUSIONS

Orthostasis, isometric handgrip exercise, and exposure to cold cause blood pressure to rise and renal arterial blood flow velocity and conductance to decrease. While these overall findings are consistent for a group, the magnitude of responses within an individual can vary from day to day. Nevertheless, these findings advance our current understanding of the magnitude and variability of vasoconstrictive effects of postural changes, exercise, and cold in humans. This approach can be used in future studies to explore genetic factors or health conditions that influence human vascular function.

## DECLARATION OF AI USE

AI tools were not used in the preparation of this manuscript.

## AUTHOR CONTRIBUTIONS

**Mohamed H. Ibrahim:** Data curation; formal analysis; investigation; methodology; software; validation; visualization. **Alison J. McLure:** Conceptualization; data curation; formal analysis; investigation; methodology; software; validation; visualization. **Mary J. Jackson:** Investigation; project administration. **Brittany Harrison:** Data curation; investigation; methodology; validation. **Sarah Brockley:** Data curation. **Elizabeth C. Jones:** Funding acquisition; methodology; supervision; validation. **A. Parker Ruhl:** Conceptualization; data curation; formal analysis; investigation; methodology; project administration; resources; supervision; validation. **Hans C. Ackerman:** Conceptualization; formal analysis; funding acquisition; investigation; methodology; project administration; resources; supervision; visualization.

## CONFLICT OF INTEREST STATEMENT

The authors declare no conflicts of interest.

## FUNDING INFORMATION

This research was supported by the Intramural Research Program of the National Institutes of Health, National Institute of Allergy and Infectious Diseases Division of Intramural Research project AI001150.

## ETHICS STATEMENT

This study complied with the Federal Policy for the Protection of Human Subjects (45 CFR, Part 36) and adhered to the principles of the Declaration of Helsinki. The clinical protocol “Collection of Human Biospecimens for Basic and Clinical Research into Globin Variants” 19‐I‐0093 was approved by the NIH Institutional Review Board (IRB) and registered at www.clinicaltrials.gov/study/NCT03937817. Screening for alpha thalassemia for some study participants was performed using a separate study 16‐I‐0065 “Screening for Alpha Thalassemia in Healthy Volunteers” which was also approved by the NIH IRB and registered at www.clinicaltrials.gov/study/NCT02692872. All study participants gave written informed consent.

## DISCLAIMERS

The contributions of the NIH authors are considered Works of the United States Government. The findings and conclusions presented in this paper are those of the authors and do not necessarily reflect the views of the NIH or the U.S. Department of Health and Human Services.

## Supporting information


Supplemental Tables 1‐4.

Supplemental Figures 1‐6.

Supplemental Doc 1.

Supplemental Doc 2.

## Data Availability

Source data for this study is available to verified researchers upon reasonable request by contacting the corresponding author.
