# Supplementary material for: Magnitude and variability of blood pressure and renal vascular conductance responses to postural changes, exercise, and cold in black adults: A pilot study
Source: Physiol Rep. 2026 May 5;14(9):e70888. doi: 10.14814/phy2.70888 (PMC13144747; doi:10.14814/phy2.70888)
Supplement: Supplementary file 1 — Supplemental Tables 1‐4. [file PHY2-14-e70888-s003.pdf]

Table S1. Blood pressure, renal arterial velocity, and renal vascular conductance responses to orthostasis, isometric handgrip exercise, and cold exposure during first visits.

	SBP (mmHg)	DBP (mmHg)	PSV (cm/s)	EDV (cm/s)	Systolic RVC (cm/s/mmHg)	Diastolic RVC (cm/s/mmHg)
**Orthostasis**						
Baseline	111.5 ± 11.7	55.3 ± 8.3				
1 min	123.4 ± 11.0<br>p = 0.0018	70.3 ± 7.0<br>p < 0.0001				
3 min	125.0 ± 10.3<br>p = 0.0055	73.2 ± 5.6<br>p < 0.0001				
**Handgrip**						
Baseline	120.5 ± 12.7	58.6 ± 5.9	115.6 ± 17.1	41.7 ± 7.1	0.9740 ± 0.16	0.7374 ± 0.17
MVE	131.2 ± 11.6<br>p = 0.0060	68.1 ± 6.3<br>p = 0.0009	103.3 ± 10.2<br>p = 0.0344	37.8 ± 6.0	0.7866 ± 0.10<br>p = 0.0103	0.5565 ± 0.11<br>p = 0.0023
30%	121.2 ± 9.1	59.3 ± 7.9	117.2 ± 20.9	38.6 ± 9.6	0.9757 ± 0.18	0.6669 ± 0.18
50%	122.6 ± 11.0	60.7 ± 8.5	111.0 ± 14.0	33.7 ± 7.0<br>p = 0.0107	0.9081 ± 0.14	0.5830 ± 0.18
70%	128.9 ± 11.6	65.2 ± 7.3<br>p = 0.0315	101.5 ± 19.6	34.1 ± 9.2<br>p = 0.0258	0.7976 ± 0.16	0.5498 ± 0.15<br>p = 0.0110
100%	136.4 ± 12.8<br>p = 0.0146	71.3 ± 9.0<br>p = 0.0156	105.1 ± 16.5<br>p = 0.0200	37.6 ± 5.6	0.7959 ± 0.12<br>p = 0.0049	0.5410 ± 0.08<br>p = 0.0332
**Cold**						
Baseline	124.6 ± 10.2	62.1 ± 12.3	125.0 ± 25.6	42.8 ± 10.5	1.012 ± 0.26	0.7338 ± 0.29
1 min	152.4 ± 8.7<br>p < 0.0001	85.8 ± 10.6<br>p < 0.0001	95.6 ± 14.6<br>p = 0.0004	34.9 ± 6.0<br>p = 0.0340	0.6166 ± 0.10<br>p = 0.0004	0.4110 ± 0.08<br>p = 0.0049
5 min	128.6 ± 9.2	64.7 ± 12.0	117.6 ± 16.7	42.9 ± 8.7	0.9151 ± 0.14	0.6929 ± 0.21
10 min	128.2 ± 11.8	65.7 ± 10.8	118.8 ± 15.6	42.7 ± 5.4	0.9195 ± 0.13	0.6687 ± 0.13

Supplemental Table 1. Blood pressure, renal arterial velocity, and renal vascular conductance responses to orthostasis, isometric handgrip exercise, and cold exposure during first visits. All data presented is mean ± standard deviation. The values in this table are plotted in figures 1-5. Statistical significance was assessed using one-way ANOVA with Dunnett's test, comparing each time point to baseline. An asterisk below a value represents statistical significance. MVE, maximum voluntary effort; mmHg, millimeter of mercury; cm/s, centimeters per second; cm/s/mmHg, centimeters per second per millimeter of mercury.

Table S2. Blood pressure, renal arterial velocity, and renal vascular conductance responses to orthostasis, isometric handgrip exercise, and cold exposure during second visits

	SBP (mmHg)	DBP (mmHg)	PSV (cm/s)	EDV (cm/s)	Systolic RVC (cm/s/mmHg)	Diastolic RVC (cm/s/mmHg)
**Orthostasis**						
Baseline	112.6 ± 12.2	55.2 ± 8.1				
1 min	121.3 ± 12.1	68.9 ± 10.1<br>p < 0.0001				
3 min	123.7 ± 10.5<br>p = 0.0443	70.9 ± 8.9<br>p < 0.0001				
**Handgrip**						
Baseline	118.6 ± 10.7	64.1 ± 12.4	109.5 ± 23.5	41.6 ± 9.0	0.9358 ± 0.25	0.6781 ± 0.23
MVE	131.8 ± 6.6<br>p = 0.0006	73.8 ± 9.6<br>p = 0.0050	103.0 ± 22.4	38.0 ± 7.9<br>p = 0.0286	0.7838 ± 0.18	0.5246 ± 0.13<br>p = 0.0119
30%	123.0 ± 11.0	65.1 ± 10.2	105.1 ± 23.0	35.5 ± 7.8<br>p = 0.0184	0.8608 ± 0.21	0.5643 ± 0.17
50%	120.9 ± 14.8	64.4 ± 7.9	109.2 ± 19.8	36.1 ± 9.2<br>p = 0.0111	0.9189 ± 0.21	0.5672 ± 0.16<br>p = 0.0164
70%	126.1 ± 9.4<br>p = 0.0322	68.4 ± 8.8	106.3 ± 21.1	34.8 ± 7.7<br>p = 0.0355	0.8534 ± 0.21	0.5175 ± 0.13<br>p = 0.0465
100%	135.4 ± 9.3<br>p = 0.0032	75.6 ± 10.0<br>p = 0.0096	100.2 ± 13.8	32.3 ± 12.7<br>p = 0.0224	0.6784 ± 0.15	0.4349 ± 0.18<br>p = 0.0065
**Cold**						
Baseline	126.2 ± 12.5	68.9 ± 9.4	105.4 ± 21.9	38.5 ± 6.5	0.8352 ± 0.23	0.5675 ± 0.12
1 min	151.9 ± 13.9<br>p = 0.0004	89.1 ± 14.8<br>p < 0.0001	94.6 ± 19.3<br>p = 0.0451	35.9 ± 6.9	0.6369 ± 0.18<br>p = 0.0023	0.4181 ± 0.12<br>p = 0.0079
5 min	127.6 ± 13.6	69.2 ± 14.1	104.2 ± 24.9	38.4 ± 8.3	0.8273 ± 0.23	0.5711 ± 0.16
10 min	127.3 ± 10.3	68.8 ± 12.9	108.8 ± 22.5	38.0 ± 9.3	0.8565 ± 0.17	0.5728 ± 0.19

Supplemental Table 2. Blood pressure, renal arterial velocity, and renal vascular conductance responses to orthostasis, isometric handgrip exercise, and cold exposure during second visits. All data presented is mean ± standard deviation. This values in this table are plotted in figures 1-5. Statistical significance was assessed using one-way ANOVA with Dunnett’s test, comparing each time point to baseline. An asterisk below a value represents statistical significance. MVE, maximum voluntary effort; mmHg, millimeter of mercury; cm/s, centimeters per second; cm/s/mmHg, centimeters per second per millimeter of mercury.

Table S3. Percent change in blood pressure, renal arterial velocity, and renal vascular conductance responses to adrenergic stimuli compared to baseline during first visits

	SBP (%)	DBP (%)	PSV (%)	EDV (%)	Systolic RVC (%)	Diastolic RVC (%)
**Orthostasis**						
Baseline	-	-				
1 min	10.7<br>p = 0.0018	27.1<br>p < 0.0001				
3 min	12.1<br>p = 0.0055	32.4<br>p < 0.0001				
**Handgrip**						
Baseline	-	-	-	-	-	-
MVE	8.9<br>p = 0.0060	22.5<br>p = 0.0009	-10.6<br>p = 0.0344	-9.4	-19.2<br>p = 0.0103	-24.5<br>p = 0.0023
30%	0.6	6.7	1.4	-7.4	0.2	-9.6
50%	1.7	9.2	-4.0	-19.2<br>p = 0.0107	-6.8	-20.9
70%	7.0	17.3<br>p = 0.0315	-12.2	-18.2<br>p = 0.0258	-18.1	-25.4<br>p = 0.0110
100%	13.2<br>p = 0.0146	28.2<br>p = 0.0156	-9.1<br>p = 0.0200	-9.8	-18.3<br>p = 0.0049	-26.6<br>p = 0.0332
**Cold**						
Baseline	-	-	-	-	-	-
1 min	22.3<br>p < 0.0001	38.2<br>p < 0.0001	-23.5<br>p = 0.0004	-18.5<br>p = 0.0340	-39.1<br>p = 0.0004	-44.0<br>p = 0.0049
5 min	3.2	4.2	-5.9	0.2	-9.6	-5.6
10 min	2.9	5.8	-5.0	-0.2	-9.1	-8.9

Supplemental Table 3. Percent change in blood pressure, renal arterial velocity, and renal vascular conductance responses to adrenergic stimuli compared to baseline during first visits. Percent changes compared to baseline were calculated as ((Response-Baseline)/(Baseline)*100). The changes presented in this table are plotted in figures 1-5. Statistical significance was assessed using one-way ANOVA with Dunnett's test, comparing each time point to baseline. An asterisk below a value represents statistical significance. MVE, maximum voluntary effort; mmHg, millimeter of mercury; cm/s, centimeters per second; cm/s/mmHg, centimeters per second per millimeter of mercury.

Table S4. Percent change in blood pressure, renal arterial velocity, and renal vascular conductance responses to adrenergic stimuli compared to baseline during second visits.

	SBP (%)	DBP (%)	PSV (%)	EDV (%)	Systolic RVC (%)	Diastolic RVC (%)
**Orthostasis**						
Baseline	-	-				
1 min	7.7	24.8<br>$p < 0.0001$				
3 min	9.9<br>$p = 0.0443$	28.4<br>$p < 0.0001$				
**Handgrip**						
Baseline	-	-	-	-	-	-
MVE	11.1<br>$p = 0.0006$	15.1<br>$p = 0.0050$	-5.9	-8.7<br>$p = 0.0286$	-16.2	-22.6<br>$p = 0.0119$
30%	3.7	1.6	-4.0	-14.7<br>$p = 0.0184$	-8.0	-16.8
50%	1.9	0.5	-0.3	-13.2<br>$p = 0.0111$	-1.8	-16.4<br>$p = 0.0164$
70%	6.3<br>$p = 0.0322$	6.7	-2.9	-16.3<br>$p = 0.0355$	-8.8	-23.7<br>$p = 0.0465$
100%	14.2<br>$p = 0.0032$	17.9<br>$p = 0.0096$	-8.5	-22.4<br>$p = 0.0224$	-27.5	-35.9<br>$p = 0.0065$
**Cold**						
Baseline	-	-	-	-	-	-
1 min	20.4<br>$p = 0.0004$	29.3<br>$p < 0.0001$	-10.2<br>$p = 0.0451$	-6.8	-23.7<br>$p = 0.0023$	-26.3<br>$p = 0.0079$
5 min	1.1	0.4	-1.1	-0.3	-0.9	0.6
10 min	0.9	-0.1	3.2	-1.3	2.6	0.9

Supplemental Table 4. Percent change in blood pressure, renal arterial velocity, and renal vascular conductance responses to adrenergic stimuli compared to baseline during second visits. Percent changes compared to baseline were calculated as ((Response-Baseline)/(Baseline)*100). The changes presented in this table are plotted in figures 1-5. Statistical significance was assessed using one-way ANOVA with Dunnett's test, comparing each time point to baseline. An asterisk below a value represents statistical significance. MVE, maximum voluntary effort; mmHg, millimeter of mercury; cm/s, centimeters per second; cm/s/mmHg, centimeters per second per millimeter of mercury.
