# Supplementary material for: Magnitude and variability of blood pressure and renal vascular conductance responses to postural changes, exercise, and cold in black adults: A pilot study
Source: Physiol Rep. 2026 May 5;14(9):e70888. doi: 10.14814/phy2.70888 (PMC13144747; doi:10.14814/phy2.70888)
Supplement: Supplementary file 2 — Supplemental Figures 1‐6. [file PHY2-14-e70888-s001.pdf]

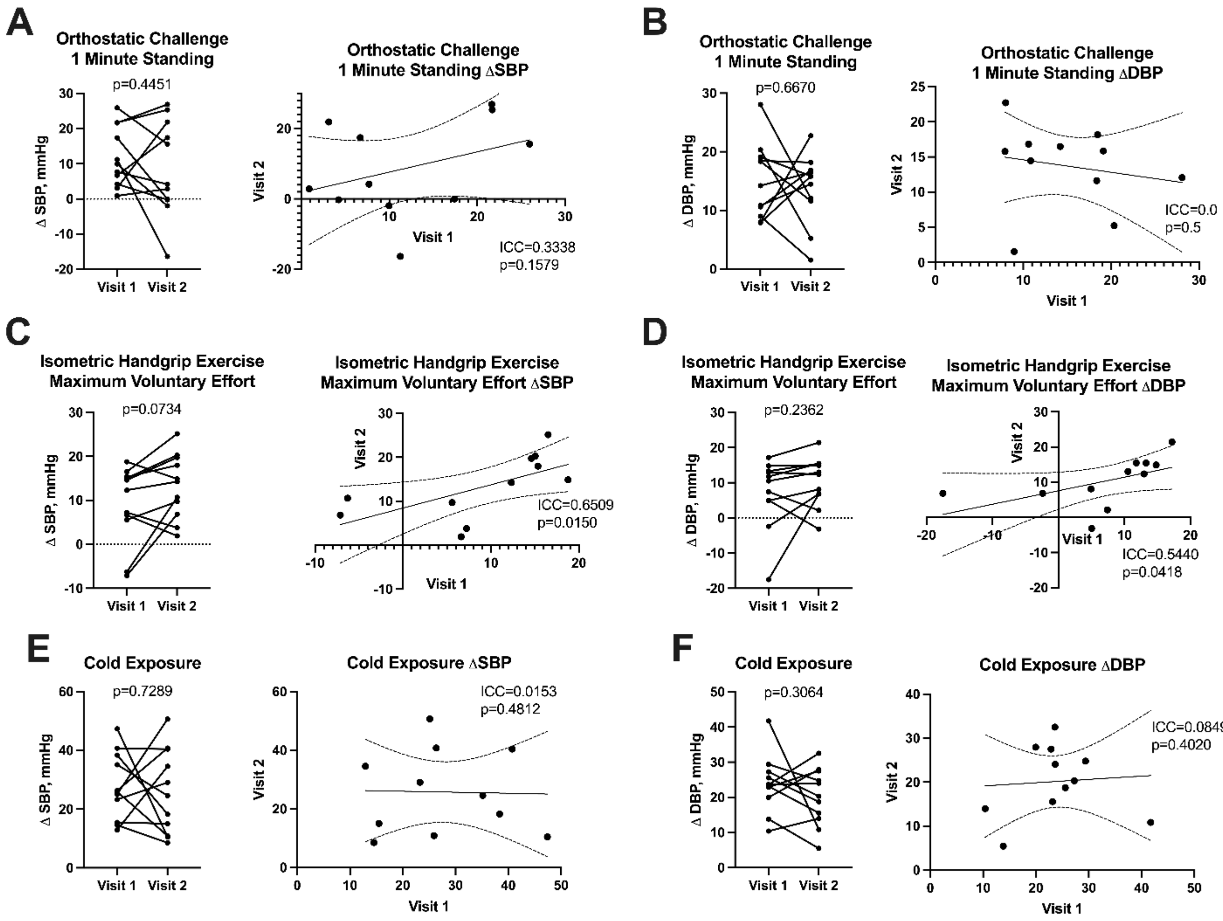

Supplemental Figure 1. Differences in blood pressure responses at 1-minute post standing, maximum voluntary effort (MVE), and cold exposure, comparing first and second visits. Changes in systolic (SBP) and diastolic blood pressure (DBP) were compared to baseline at specific points of the three adrenergic stimuli. Each line represents a single subject and connects their responses on visit one and visit two. Each point on the x/y plot represents the responses from visit one and visit two for a single subject. (A) Orthostatic challenge: The change in SBP at 1-minute standing compared to baseline from visit one to visit two. (B) Orthostatic challenge: The change in DBP at 1-minute standing compared to baseline from visit one to visit two. (C) Isometric handgrip exercise: The change in SBP at MVE compared to baseline from visit one to visit two. (D) Isometric handgrip exercise: The change in DBP at MVE compared to baseline from visit one to visit two. (E) Cold exposure: the change in SBP following one minute exposure to cold compared to baseline from visit one to visit two. (F) Cold exposure: the change in DBP following one minute exposure to cold compared to baseline from visit one to visit two. Statistical difference was assessed using paired t-test and intraclass correlation coefficients. mmHg, Millimeters of Mercury; MVE, Maximum Voluntary Effort; ICC, Intraclass Correlation Coefficient.

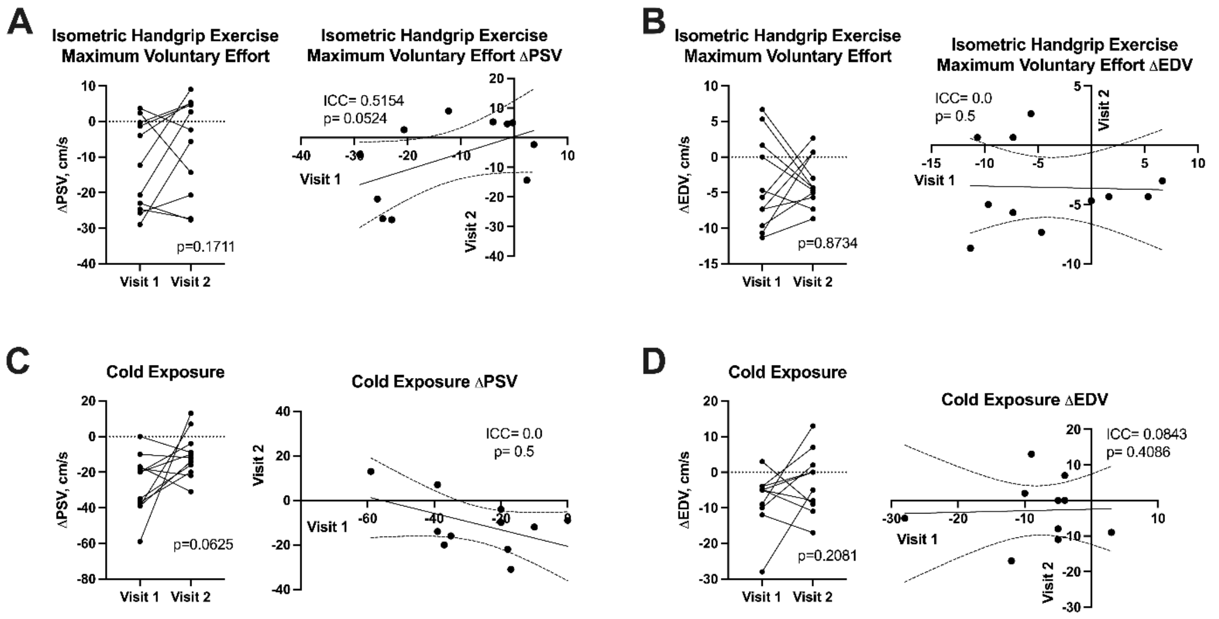

Supplemental Figure 2. Differences in renal arterial peak systolic (PSV) and end diastolic (EDV) velocities at maximum voluntary contraction (MVE) and cold exposure comparing first and second visits. Exercise and cold-induced changes in peak systolic and end diastolic renal artery velocities were compared between visits one and two. Each line represents a single subject and connects their responses on visit one and visit two. Each point on the x/y plot represents the responses from visit one and visit two for a single subject. (A) Isometric handgrip exercise: The change in PSV at MVE compared to baseline from visit one to visit two. (B) Isometric handgrip exercise: The change in EDV at MVE compared to baseline from visit one to visit two. (C) The change in PSV following one minute exposure to cold compared to baseline from visit one to visit two. (D) The change in EDV following one minute exposure to cold compared to baseline from visit one to visit two. Statistical difference between day one and day two was assessed using paired t-test. cm/s, centimeters per second; MVE, Maximum Voluntary Contraction; ICC, Intraclass Correlation Coefficient.

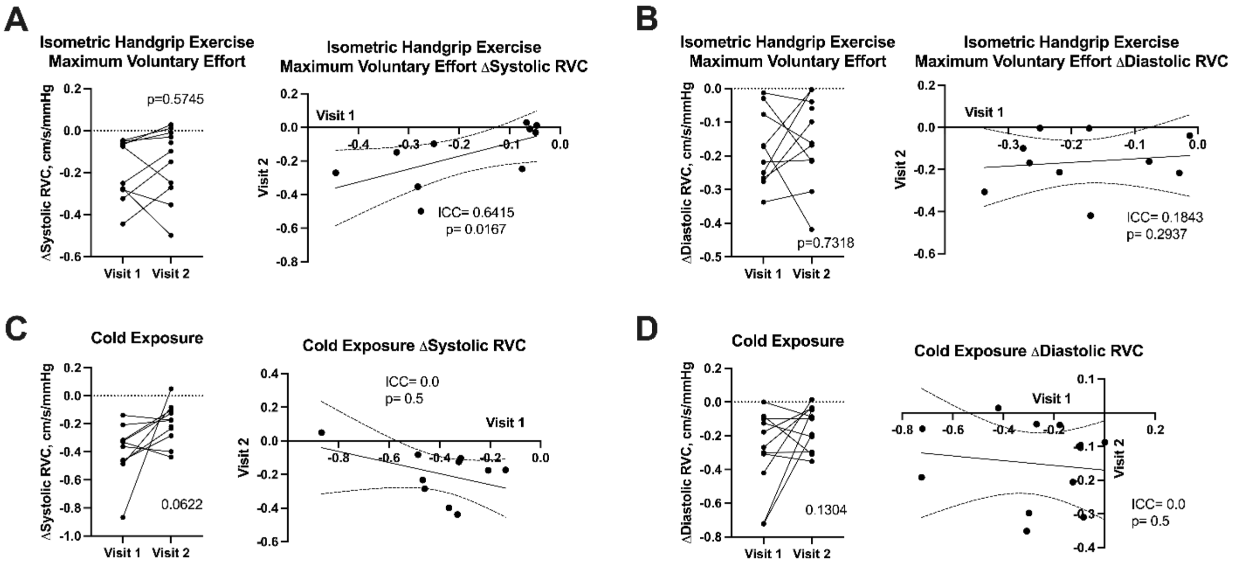

Supplemental Figure 3. Changes in renal vascular conductance (RVC) in response to cold exposure or hand grip exercise at first and second visits. Changes in RVC relative to baseline were compared at first and second visits. Each line represents a single subject and connects their responses on visit one and visit two. Each point on the x/y plot represents the responses from visit one and visit two for a single subject. (A) Isometric handgrip exercise: The change in RVC at MVE compared to baseline from visit one to visit two. (B) Isometric handgrip exercise: The change in diastolic RVC at MVE compared to baseline from visit one to visit two. (C) The change in systolic RVC following one minute exposure to cold compared to baseline from visit one to visit two. (D) The change in diastolic RVC following one minute exposure to cold compared to baseline from visit one to visit two. Statistical difference between day one and day two was assessed using paired t-test. cm/s/mmHg, centimeters per second per millimeter of mercury; MVE= Maximum Voluntary Effort; RVC, Renal Vascular Conductance; ICC, Intraclass Correlation Coefficient.

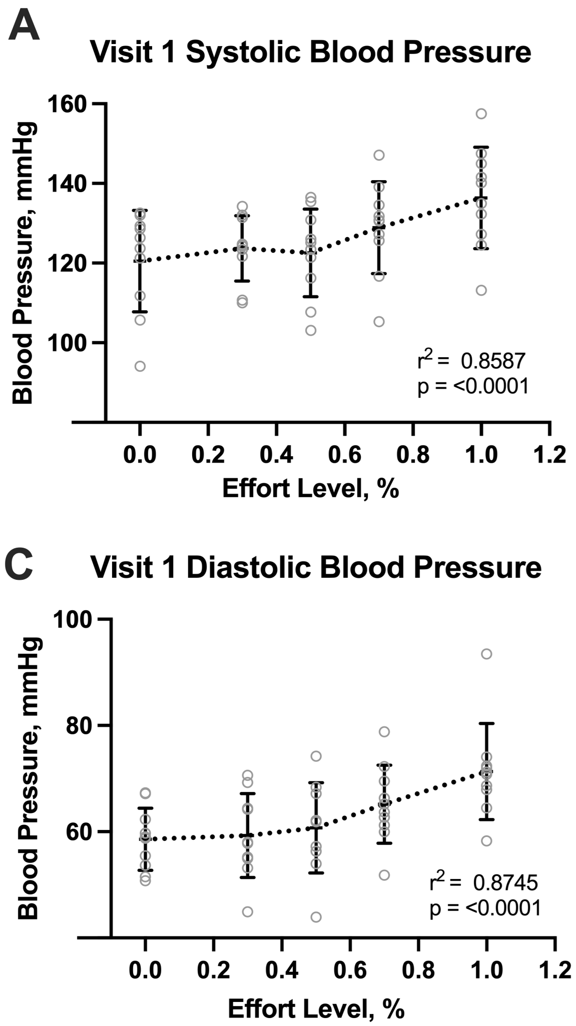

Supplemental Figure 4. Trends in systolic and diastolic blood pressures with increased effort during isometric handgrip exercise. Changes in SBP and DBP during isometric handgrip exercise from baseline to 30%, 50%, 70%, and finally 100% effort. Each point represents mean BP and error bars represent standard deviation. (A) The change in SBP with increasing effort level on visit one. (B) The change in DBP with increasing effort level on visit one. (C) The change in SBP with increasing effort level on visit two. (D) The change in DBP with increasing effort level on visit two. Repeated-measures one-way ANOVA test for linear trend was performed to calculate r^2 and statistical significance of BP trends across effort levels. mmHg, millimeter of mercury.

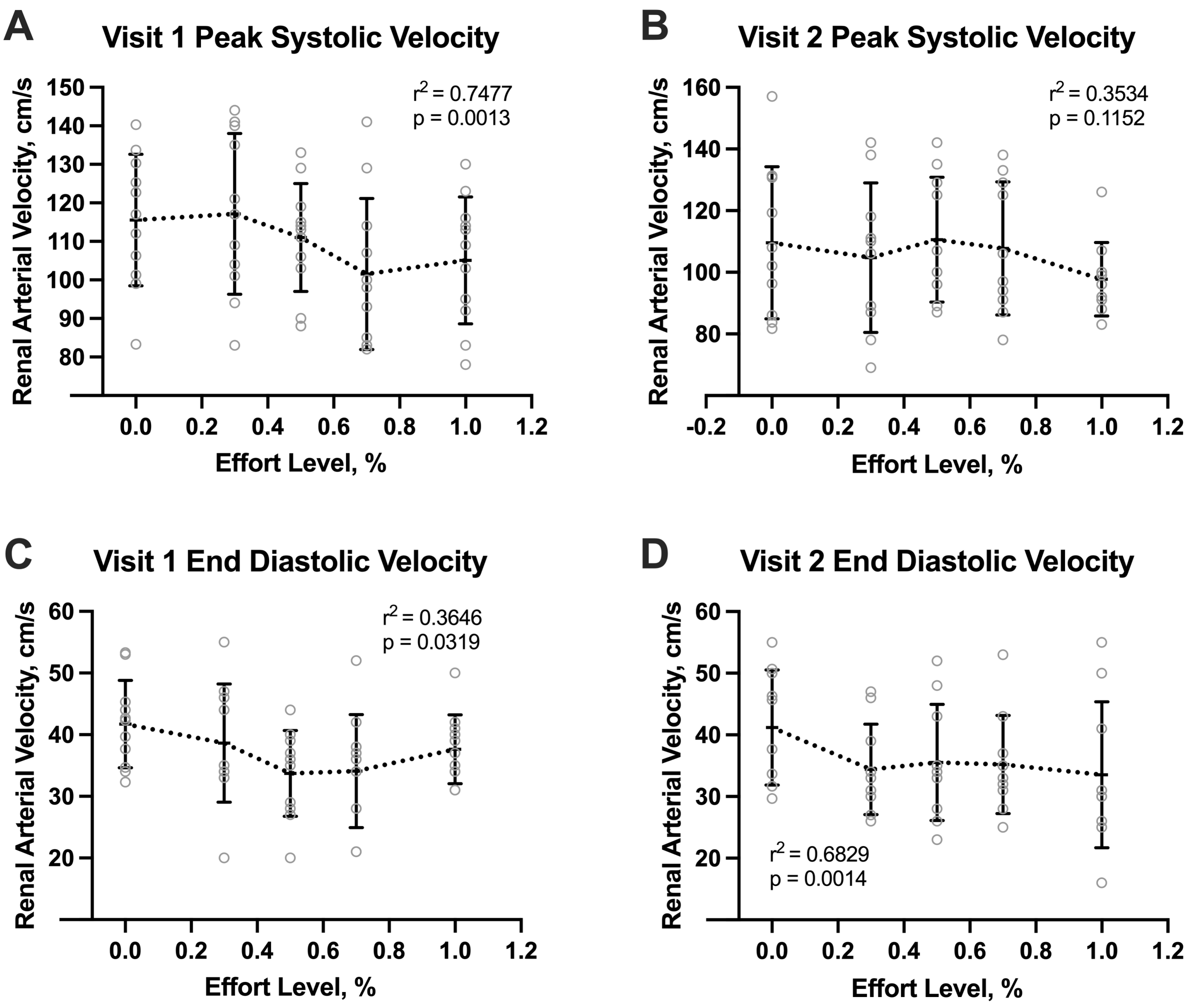

Supplemental Figure 5. Changes in peak systolic (PSV) and end diastolic (EDV) velocity with increase in effort during isometric handgrip exercise on visit one and visit two. Changes in PSV and EDV during isometric handgrip exercise from baseline to 30%, 50%, 70%, and finally 100% effort. Each point represents mean renal arterial velocity and error bars represent standard deviation. (A) The change in PSV with increasing effort level on visit one. (B) The change in EDV with increasing effort level on visit one. (C) The change in PSV with increasing effort level on visit two. (D) The change in EDV with increasing effort level on visit two. Repeated-measures one-way ANOVA test for linear trend was performed to calculate r^2 and statistical significance of BP trends across effort levels. cm/s, centimeters per second.

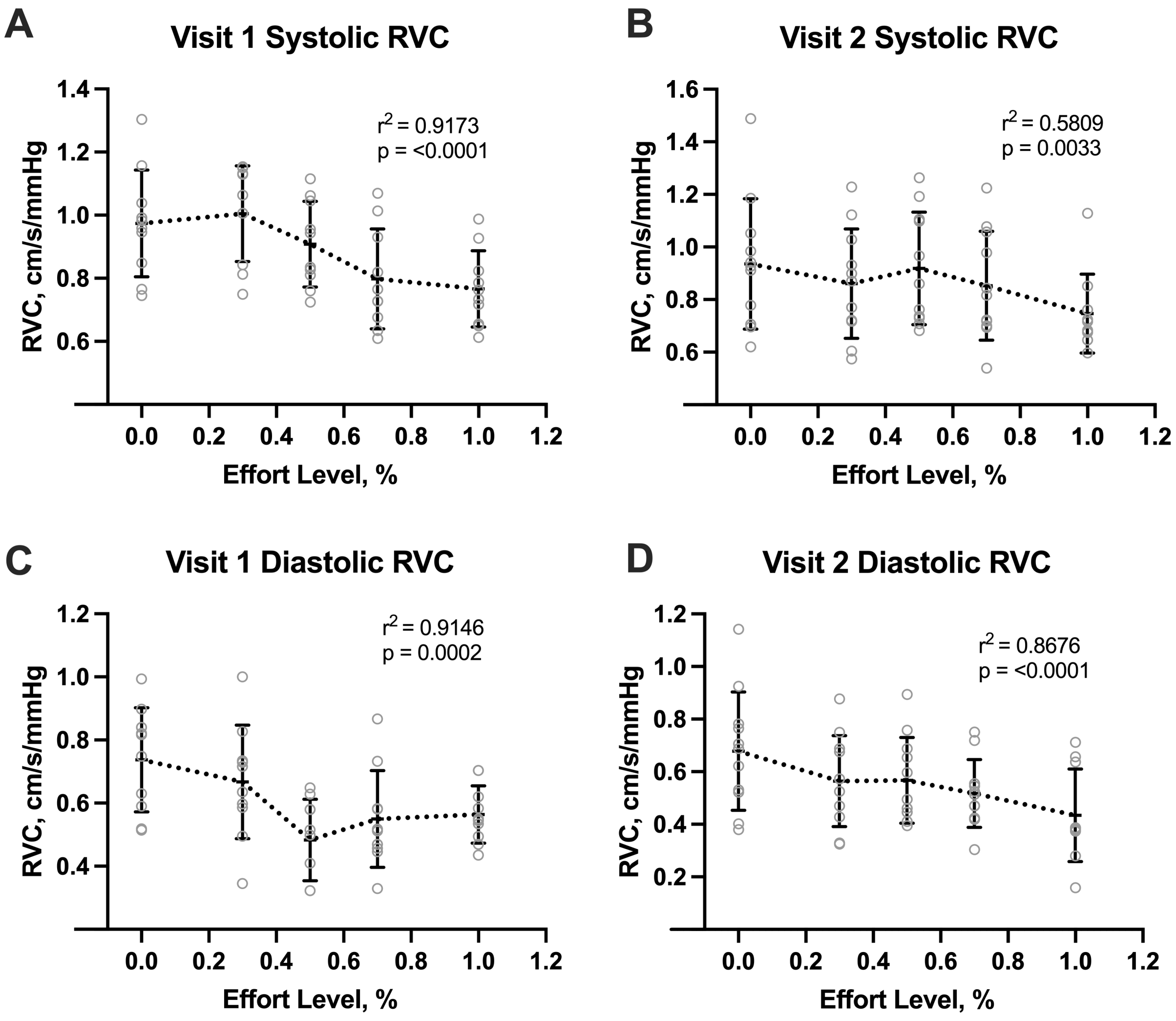

Supplemental Figure 6. Changes in systolic and diastolic renal vascular conductance (RVC) with increase in effort during isometric handgrip exercise on visit one and visit two. Changes in systolic and diastolic RVC during isometric handgrip exercise from baseline to 30%, 50%, 70%, and finally 100% effort. Each point represents mean RVC and error bars represent standard deviation. (A) The change in systolic RVC with increasing effort level on visit one. (B) The change in diastolic RVC with increasing effort level on visit one. (C) The change in systolic RVC with increasing effort level on visit two. (D) The change in diastolic RVC with increasing effort level on visit two. Repeated-measures one-way ANOVA test for linear trend was performed to calculate r^2 and statistical significance of BP trends across effort levels. cm/s/mmHg, centimeters per second per millimeter of mercury; RVC, Renal Vascular Conductance
