# Supplementary material for: Magnitude and variability of blood pressure and renal vascular conductance responses to postural changes, exercise, and cold in black adults: A pilot study
Source: Physiol Rep. 2026 May 5;14(9):e70888. doi: 10.14814/phy2.70888 (PMC13144747; doi:10.14814/phy2.70888)
Supplement: Supplementary file 3 — Supplemental Doc 1. [file PHY2-14-e70888-s004.pdf]

Revision History		
**Version No.**	**Effective Date**	**Description**
**3**	August 18th, 2025	**Minor edits to instructional language, addition of team member**
**2**	March 21st, 2024	**SOP detailing procedures for Non-Invasive Blood Pressure and Vascular Measurements in the NIH Clinical Center**
**1**	October 16th, 2023	**Assay Development SOP detailing procedures for Non-Invasive Blood Pressure and Vascular Measurements in the NIH Clinical Center**

Written by Alison McLure (IRTA), Mohamed Ibrahim, (IRTA), Parker Ruhl, MD (Staff Clinician), and reviewed by Hans Ackerman MD, DPhil, MSc (Chief, Physiology Unit).

Approved For Use For Protocol 19-I-0093 By: **Date:**

AMY P. RUHL -S Digitally signed by AMY P. RUHL - S
Date: 2025.08.18 12:18:52 -04'00'

________________________________ ____________________

A. Parker Ruhl, MD, Staff Clinician
(Protocol 19-I-0093)

1 Purpose

This SOP explains the steps necessary for administering non-invasive vascular measurements and adrenergic vasoconstrictive stimuli at the NIH Clinical Center. Standard procedures for the three adrenergic stimuli and adequate set up for each instrument is necessary for accurate data and blood pressure readings. Furthermore, instruments should be handled with care to prevent them from breaking, as well as prevent accidental injury to research participants.

2 Scope and Responsibilities

Instruction: This describes to whom or under what circumstances the document applies.

2.1 Scope:

2.1.1 The intent of this SOP is to:

- Ensure correct set up of AD Instruments’ NIBP Nano Device.
- Prevent injuries to healthy volunteers or accidental misuse of instruments due to incorrect set up.
- Ensure accurate administration of adrenergic stimuli and collection of vascular measurement data.

3 Definitions/Acronyms

3.1 NIBP Nano = Non-Invasive Blood Pressure Nano Interface

4 Set up for NIBP Nano Vascular Measurement Technique

4.1 **Room.**

4.1.1 Record room temperature at the start of set up. (Ideal temperature $\mathbf{22-26^{o}C.}$**)**

4.2 **Table/ Exam Chair/ Bed.**

4.2.1 If there is a sanitary paper sheet, rip a new sheet for each participant.

4.2.2 If there is an exam chair, position the chair to be flat.

4.2.3 Supply the participant with clean pillows, if necessary.

4.2.4 If there is a bed, ensure that sheets are tucked in and blankets are removed.

4.3 **Cold Pressor Bucket.**

4.3.1 Prepare a large bucket and fill it half with ice and half with cold water. **Ensure you have an additional small cooler with ice available.**

4.3.2 Place aquarium thermometer suction with probe into the bucket and place thermometer’s screen next to the bucket where visible.

4.3.3 Check the water temperature to ensure that it is between **$3^{o}C$ and $5^{o}C$.** Add smalls increment of ice if temperature rises above $5^{o}C$. Remove the ice from the cold water prior to inserting the subject's hand.

4.4 **Laptop connection.**

4.4.1 Ensure that the laptop charger (black with HP logo) and ethernet cable are attached.

4.4.2 Sign in with PIV card.

4.5 **NIBP Nano Device Connection.**

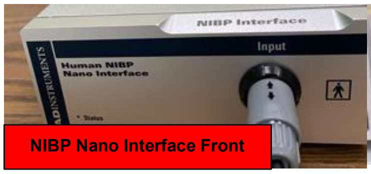

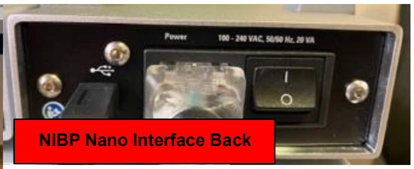

4.5.1 Using the thinnest black cord labeled **H2**, plug USB side into the laptop and plug the other side into the back of the NIBP Nano interface. Using the thick black cord labeled **H7**, plug the NIBP device into a wall outlet.

4.5.2 Take out black wrist unit from NIBP's box and plug it into the front of the NIBP Nano interface with the arrow at the top.

4.6 **Bridge Amp and Powerlab Connection.**

4.6.1 Plug beige cord labeled **H5** into the USB symbol port of the Powerlab device and the other side into the USB port of the laptop.

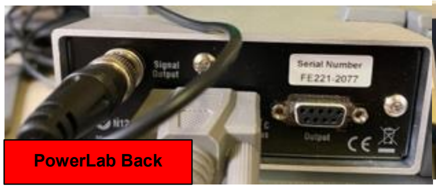

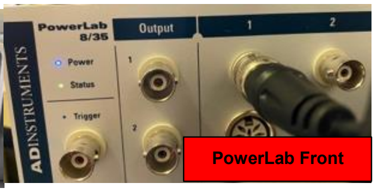

4.6.2 Plug the black BNC cable labeled **H3** into the back side of the bridge amp, twisting to secure and the other side of the cord into the front of the Powerlab in **Channel 1**, also twisting to secure.

4.6.3 Plug the thick black power cord labeled **H4** into the back of the Powerlab and the other side into an outlet.

4.6.4 Plug the handgrip cord labeled **H1** into the front of the bridge amp under input with the screw facing up and the other side into the handgrip dynamometer, ensuring that the red dots line up.

4.6.5 Confirm setup with below images:

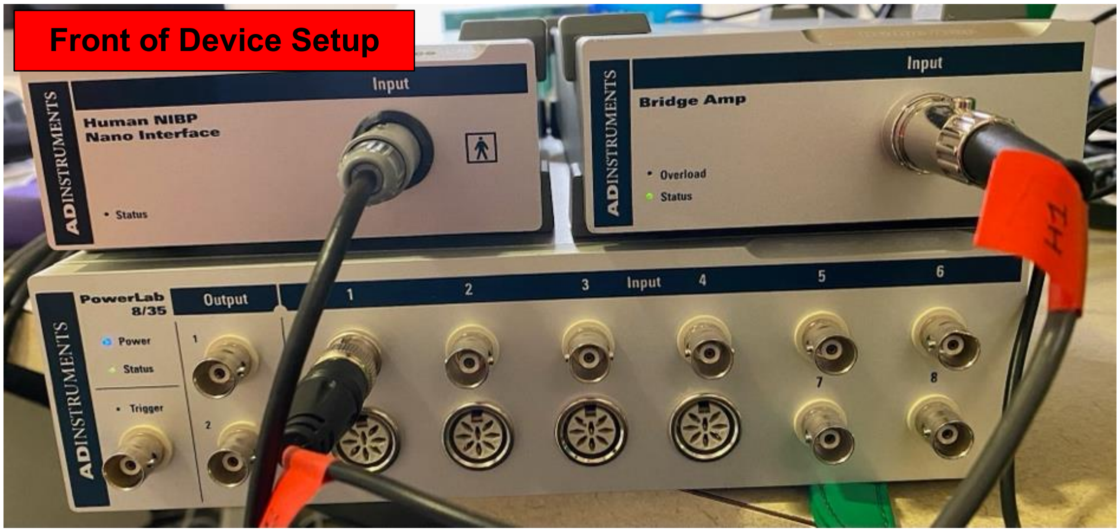

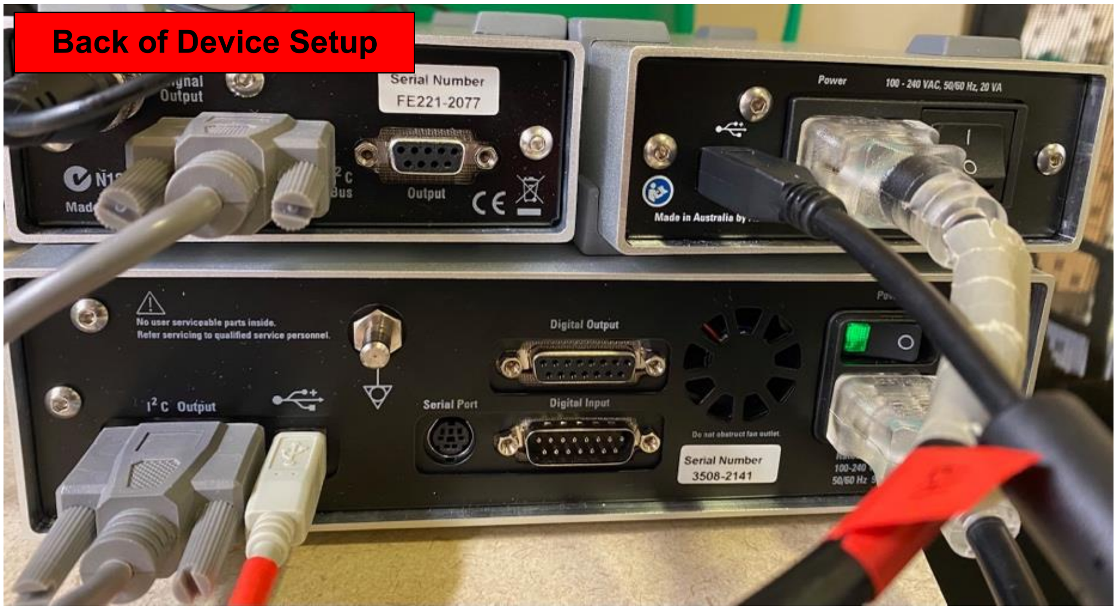

4.6.6 Switch on the Powerlab and NIBP devices.

5 Research Volunteer Check-in

5.1 Interview and Research Volunteer Teaching

5.1.1 Ask about volume of fluid intake from the night before, fasting times, and medications taken, if any.

5.1.1.1 Ask the subject if they have refrained from taking any food since midnight and otherwise ingested approximately 16 oz. (two full 8 oz. glasses) of plain water during the approximately 12-hour period prior to the study visit.

5.1.1.2 Ask the subject if they have taken any of the following medications during the last 12 hours:

NSAIDS (non-steroidal anti-inflammatory drugs) (e.g., aspirin, ibuprofen [Motrin, Advil], naproxen [Aleve] or celecoxib [Celebrex])

Caffeine (e.g., coffee, tea, green tea, soda, or energy drinks)

Decongestant medications (e.g., pseudoephedrine [Sudafed], Mucinex-D, Claritin-D, Allegra-D, phenylephrine [Sudafed PE], Mucinex Sinus Max, Nyquil Severe Cold and Flu, Nyquil/Dayquil Congestion Relief or any store-brand medications that contain phenylephrine)

Dietary supplements with more than 1 gram of L-arginine or L-citrulline or supplements containing either (e.g., Herbalife Niteworks)

Phosphodiesterase inhibitors (e.g., sildenafil [Viagra], Cialis, Levitra)

5.1.1.3 Ask the subject if they have used any nicotine and marijuana products for 30 days prior to the visit.

5.1.2 Ask subject to empty their bladder, collecting a small urine sample in a sterile cup.

5.1.2.1 The subject should be instructed to follow the “clean catch method” for urine collection and collect their urine sample midstream, fill the cup halfway, and finish their urination in the toilet.

5.2 Measure urine specific gravity using OPTIKA HR-160N Hand Refractometer.

5.2.1 With sterile gloves, using a clean surface, use a transfer pipette to place 2-3 drops of subject’s urine onto the LED daylight plate of the specific gravity refractometry (OPTIKA, Italy) device.

5.2.2 Gently close the plate’s flap, aim the daylight plate toward a light source, and look into the eyepiece to read result lines and record specific gravity.

5.2.2.1 If greater than 1.035 ug, then instruct the subject to drink an additional 8 oz of water.

5.2.3 Clean LED daylight plate with clinic issued disinfecting wipes and allow to dry.

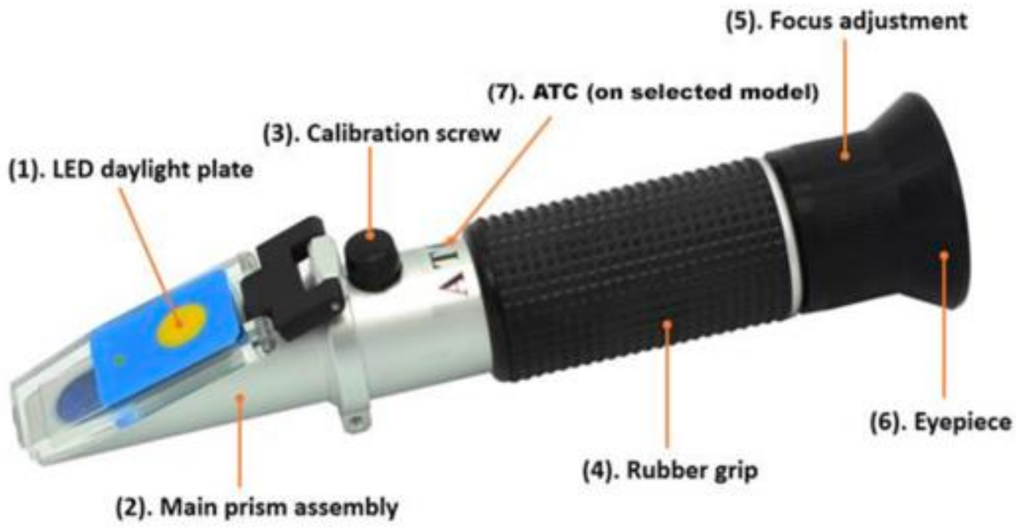

5.3 Protocolized Blood Pressure Measurements

5.3.1 Ask subject to remove jewelry or items on their arms and hands.

5.3.2 Instruct the subject to sit in a chair with their feet resting on the floor and place their arms on the arm rests.

5.3.3 Proceed to take protocolized blood pressure measurements in both arms and record these values in crimson.

5.3.4 Prior to setting up NIBP Nano Device on subject, offer the subject small amounts of (no more than 8 oz.) water to drink to maintain adequate hydration level.

5.4 **Labchart.**

5.4.1 Click the Labchart icon on the desktop. The pop-up window should verify that the application recognizes the Powerlab device and NIBP device.

5.4.2 Ensure the status light on the Powerlab and bridge amp box is green.

5.4.3 Once recognized, open the settings file within lab chart titled “NIBP with Renal Ultrasound_DATE”. Ensure green light next to status lights up on the NIBP.

5.4.4 To ensure this session will be saved, click “Save as.”

5.4.5 Name the file “AB0XXX_YYYYMMDD”.

5.4.6 And click "Save" after each stimulus and throughout the session to ensure all data is saved.

5.5 **Set up for NIBP Nano Device.**

5.5.1 Measure the subject's finger cuff size on the non-dominant hand.

5.5.1.1 Place their middle finger next to the measurement device under the angled side.

5.5.1.2 Wrap the white strap around middle phalanx of the finger above the knuckle and pull the excess on top of the measurement device.

5.5.1.3 Where the end of the strap reaches within the small, medium, and large range, indicates the cuff size needed.

5.5.1.4 If in between sizes, chose the smaller size.

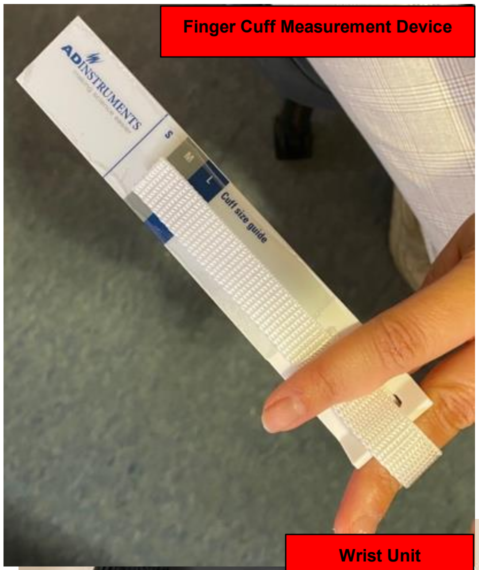

5.5.2 Plug the chosen finger cuffs into the wrist unit as per image on the right.

5.5.2.1 Place the first cuff's silver adapter into **C1**, with the red line facing upwards. Plug the clear air tube tightly on the right of the silder adapter.

5.5.2.2 Place the second cuff's silver adapter into **C2**, with the red line facing upwards. Plug the clear air tube tightly on the left of the silder adapter.

5.5.2.3 Place the HCU's silver adapter in the center hole of the wrist unit with the red line facing upwards.

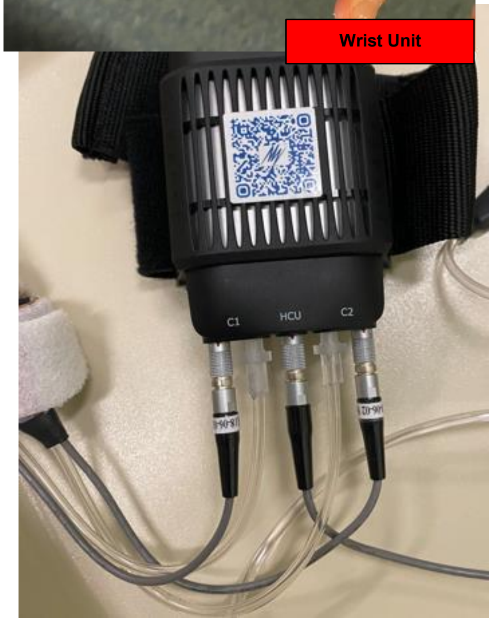

5.5.3 Strap the wrist unit around the subject's wrist so that it fits snug, but not uncomfortable.

5.5.4 Place the C1's finger cuff on the middle finger of the non-dominant hand.

5.5.4.1 Gently open the cuff enough to slide the finger through.

5.5.4.2 The two grey dots on the inside of the cuff should be on the palmer surface of the finger and the cuff's cord should cross the palmer surface of the hand as well.

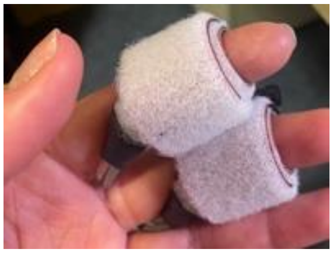

5.5.4.3 Wrap the cuffs so that they do not occlude blood flow to the finger.

5.5.5 Place the C2 finger cuff on the pointer figure using steps 5.5.4.1 – 5.5.4.3.

5.5.6 Place the two leads of the height correction unit together so they touch and calibrate the HCU.

5.5.6.1 On lab chart, click setup -> Human NIBP and HCU test -> zero -> OK.

5.5.6.2 If you remove the NIBP or pause data collection, you must zero the HCU unit each time.

5.5.7 Use prepared EKG and Velcro sticker and place it onto the subject's chest at the right atrium.

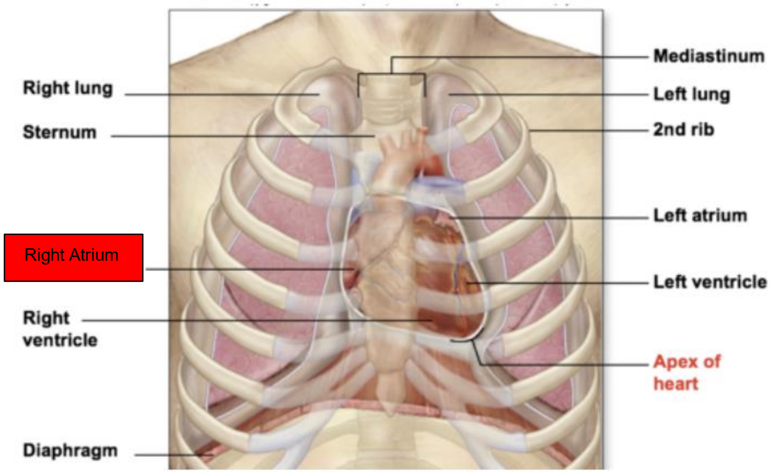

5.5.8 Velcro the rectangular part of the HCU on the middle finger and the round part of the HCU on the Velcro spot placed on the subject's chest just to the right of the sternum between the third and fourth rib, feeding the clear tube underneath their shirt. Use skin-safe tape to secure the HCU unit to the chest.

6 Orthostatic Blood Pressure Measurement SOP, BP Procedure:

6.1 Press start on the Lab chart application if not already running and allow NIBP Nano quality level to reach 9 or 10 while subject is laying supine, in a relaxed state with their legs uncrossed.

6.2 Allow continuous readings for 5 minutes while in supine position (quality level should go to 9 or 10 during these 5 minutes, if not, continue until adequate quality level is reached).

6.2.1 Press F1 after the 5 minutes to label this reading as “Start of Baseline - Supine” and press F2 after one minute has passed to label “End of Baseline -Supine”

6.3 Instruct the subject to transition from a supine position to a standing position in a controlled way ensuring they do not put pressure on the finger cuffs, with arms by their side, and not leaning on the bed once standing. Mark when they start the transition by pressing “F3” to label as “Transition – supine to standing” and press “F4” to mark when they reach the standing position as “Start of Standing.”

6.3.1 Monitor the subject for any signs of pallor, diaphoresis, and syncope.

6.4 Allow NIBP Nano to measure for 1 minute and press “F5” to label this reading as “End of Standing - 1 min”.

6.5 Allow NIBP Nano to measure for an additional 2 minutes and press “F6” to label this reading as “End of Standing - 3 min”.

6.6 Mark the end of the stimuli by pressing “F7” to label as “End of stimuli” and instruct subject to return to a supine position for 5 minutes.

6.7 Press save before proceeding to the next stimuli and press “stop” in between sessions.

7 Handgrip Exercise with Renal Artery Ultrasound SOP, NIBP Nano Procedure:

7.1 Ask the subject if they would like to empty their bladder in between sessions needed **recalibrate the HCU unit if they take off the NIBP cuff”**. They may continue to take small sips of water to remain hydrated.

7.1.1 Instruct the subject to lay supine on the bed with abdomen exposed.

7.1.2 Allow 15-20 minutes for sonographer to conduct their examination.

7.1.3 Continue renal ultrasound measurements for the right kidney through one handgrip exercises and cold exposure stimuli. To relax the abdomen, place a towel under the subjects knees and feet.

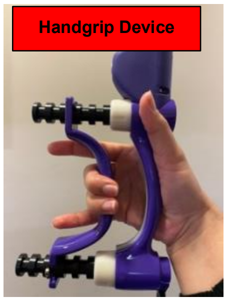

7.2 **Set up Handgrip dynamometer:**

7.2.1 Instruct the subject to insert their dominant hand in the handgrip's black loop, then have them curl their fingers to a 90-degree angle and place the purple dynamometer in their hand with the bulky side up. See image to the right for correct positioning of the hand.

7.2.2 Ensure the subjects curled fingers fit snuggly over the dynamometer handle and that their arm is horizontal.

7.2.2.1 If they cannot wrap around the dynamometer with their fingers in a 90-degree angle, then adjust the dynamometer's gap to be smaller.

7.2.2.2 If there is space between their curled fingers and the dynamometer, then adjust the dynamometer's gap to be bigger ->

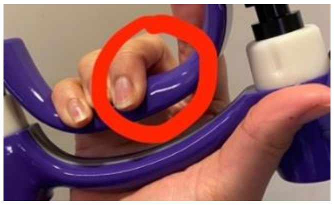

7.3 Press start on the labchart software if recording has paused and tell the subject they will feel some pulsing in their middle finger, but to let us know if it uncomfortable.

7.4 Begin the renal artery ultrasound from an anterior position, ensuring ultrasound gel is warmed between 95-105 degrees F.

7.5 Ensure quality level on lab chart software is at 9 or 10 before starting baseline recording.

7.6 Collect renal blood flow dynamics and nano blood pressure measurements for baseline measurements to be collected.

7.6.1 In order to coordinate the timing of renal ultrasound image capture with the NIBP device, the NIBP device operator should say "start" at the start of the effort. After 15 seconds, the operator should say "image." After 3-5 more seconds, the operator shoud say "relax" to instruct the subject to stop the effort.

7.6.2 To mark these data points, press "F8" to label this reading as "Start of Baseline 1 - Handgrip" at "start" verbal cue and after 15 seconds press "F9" to "End of Baseline 1 – Handgrip" at "image" cue. **Ensure that you time the end of the 15 seconds with the ultrasound technician to ensure accurate timing. Repeat these steps for every effort.**

7.6.3 Repeat baseline measurements for the handgrip stimuli three times with one-minute rest in-between.

7.6.3.1 Press “F10” for “Start of Baseline 2 - Handgrip” and “F11” for “End of Baseline 2 – Handgrip”

7.6.3.2 Press “F12 for “Start of Baseline 3 – Handgrip” and “Shift+A” for “End of Baseline 3 – Handgrip”.

7.7 **Determine the subject’s maximum voluntary contraction (MVC)**

7.7.1 Enlarge the raw force data channel.

7.7.2 Instruct the subject to squeeze the dynamometer as hard as possible for 15 seconds then, take a break for 1-minute, and repeat this cycle of maximal efforts and breaks for a total of 3 times.

7.7.2.1 Press “Shift+B” at the beginning of the first 15-second contraction to label the reading as “MVC Session 1 Start”. “Press “Shift+C” to label “MVC Session 1 End”

7.7.2.2 Press “Shift+D” at the beginning of the first 15-second contraction to label the reading as “MVC Session 2 Start”. “Press “Shift+E” to label “MVC Session 2 End”

7.7.2.3 Press “Shift+F” at the beginning of the first 15-second contraction to label the reading as “MVC Session 3 Start”. “Press “Shift+G” to label “MVC Session 3 End”

7.7.3 Save and press “Stop” the LabChart software and note which of the three maximal efforts is highest. Use the comment feature to note the average force over that 15 second contraction.

7.7.4 Pause the recording and enter this number into the denominator of the arithmetic value within each of the 30, 50, 70, and 100% channels.

7.8 Press the “Start” button again and continue to collect renal blood flow dynamics and nano blood pressure measurements until the quality level reaches 9 or 10.

7.9 Instruct the subject to squeeze the handgrip dynameter for 15 seconds increments at 30%, 50%, 70%, and 100% of their MVC with 1 minute rest periods in between each bout of exercise.

7.9.1 Coach each participant to steadily squeeze the handgrip dynamometer.

7.9.2 Each participant is allowed to have a practice squeeze in between each contraction effort. Ensure that they have a 1-minute rest after any contraction effort.

7.9.3 For each 15-second effort, label the LabChart graph by pressing “Shift+H” for “30% Start”, “Shift+I” for “30% End”, “Shift+J” for “50% Start”, “Shift+K” for “50% End”, “Shift+L” for “70% Start”, “Shift+M” for “70% End, “Shift+N” for “100% Start”, and “Shift+O” for “100% End”.

7.10 Press save before proceeding to the next stimuli and press “stop” to pause the labchart readings.

8 Cold Pressor Test SOP, BP Procedure:

8.1 Ensure that the subject empties their bladder in between sessions if need be.

8.2 Press “Start” and allow recordings to continue for 5 minutes and ensure quality level on lab chart software is at 9 or 10 before starting baseline recording.

8.3 After quality level is reached, press “Shift+P” to label this reading as “Start of Baseline - Cold Exposure”, wait 15 seconds and press “Shift+Q” to label the “End of Baseline - Cold Exposure”. Ensure to time the beginning and end of each effort correctly with the ultrasound technician.

8.4 Instruct the subject to place their dominant arm in the cold temperature bath (from fingertips to styloid process of the wrist) for 60 seconds.

8.4.1 Press “Shift+R” to label this reading as “Start of Cold Exposure”.

8.4.2 After 1-minute, press “Shift+S” to label the “End of Cold Exposure”.

8.5 Instruct the subject to remove their arm after exposure and place on a towel. Dry the subject’s arm and instruct them to relax in a supine position for 10 minutes.

8.6 5 minutes after the cold exposure ends, press “Shift+T” to label this reading as “Start of 5 min post-cold exposure”. Wait 45 seconds and press “Shift+U” for “End of 5 min post-cold exposure”.

8.7 Allow for 5 more minutes of recording (10 minutes after cold exposure). Press “Shfit+V” to label this reading as “Start of 10 min post-cold exposure”. Wait 45 seconds and press “Shift+W” for “End of 10 min post-cold exposure”.

8.8 Press “Save” and then press “Stop” to end session.

9 Bank of cords, devices, and comment short cuts:

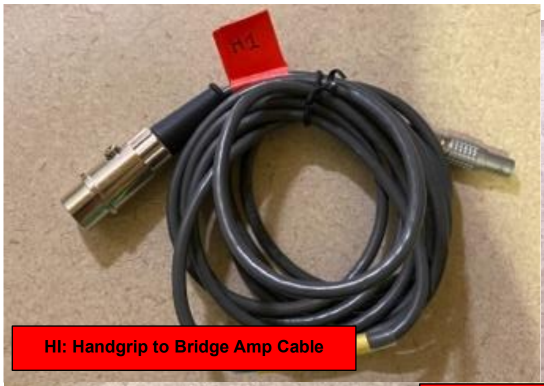

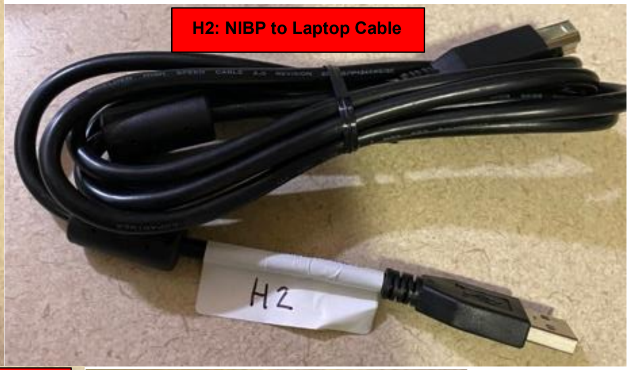

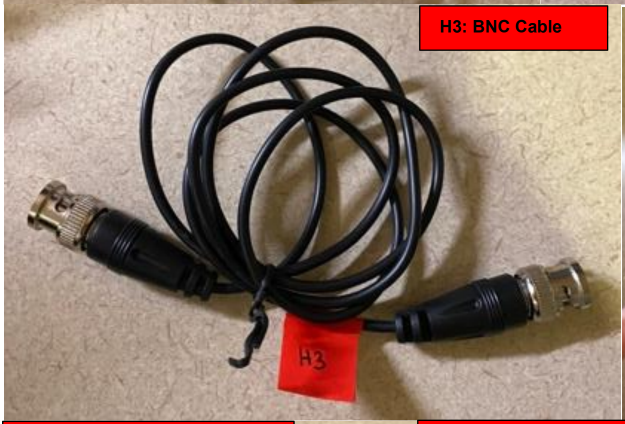

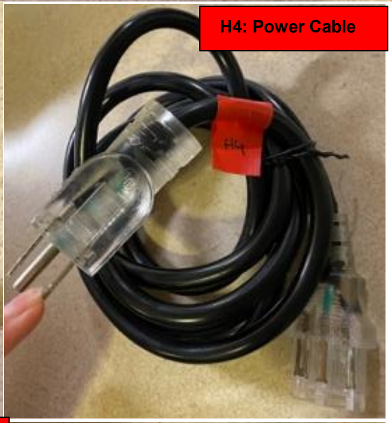

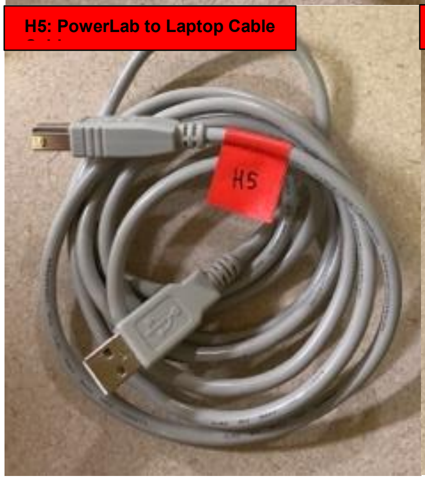

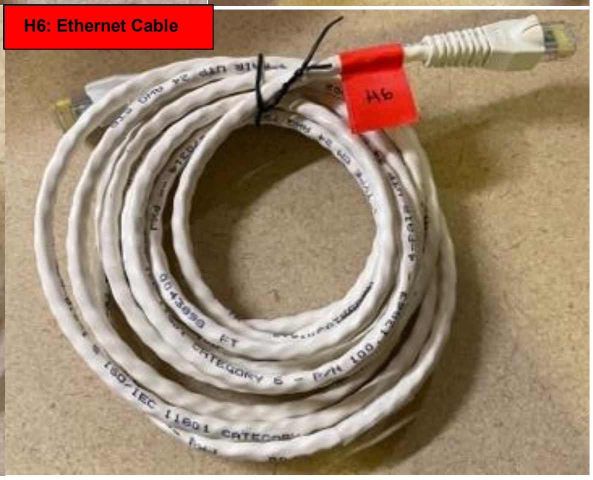

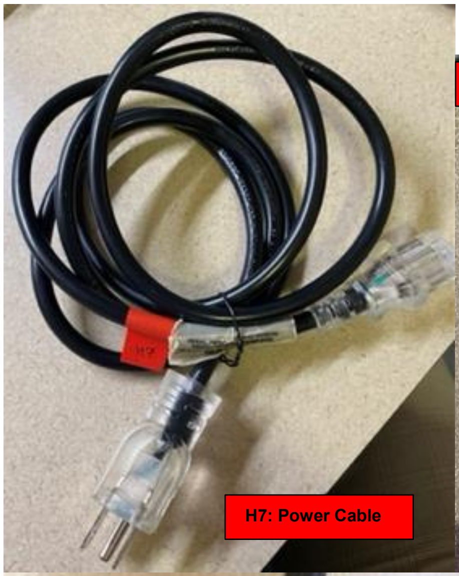

H7: Power Cable

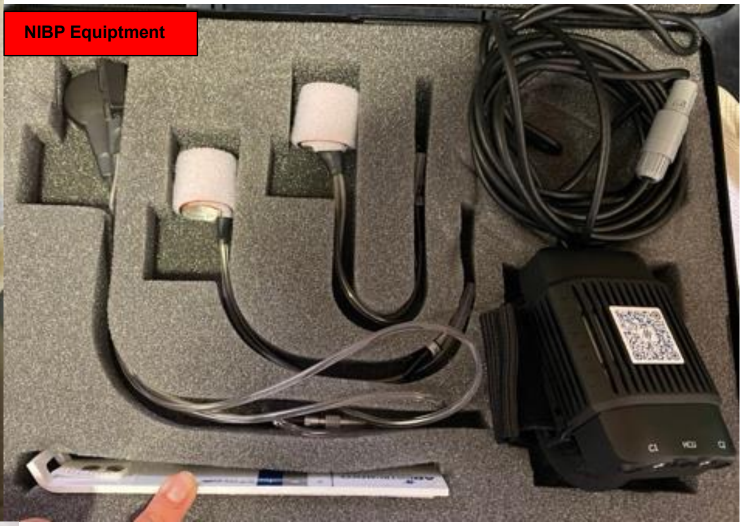

NIBP Equiptment

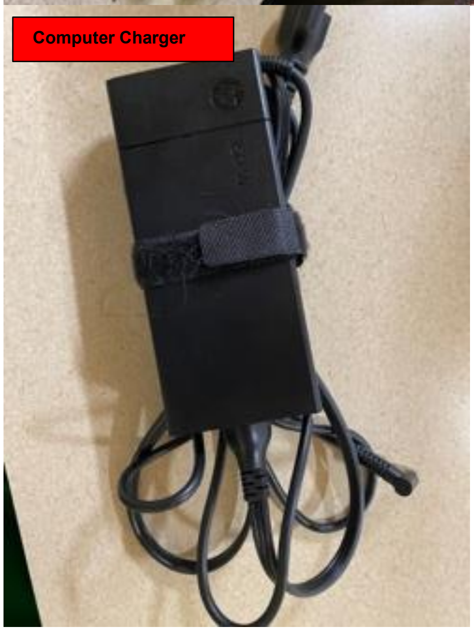

Computer Charger

10 Table of Shortcuts

Key	Comments
F1	Start of Baseline - Supine
F2	End of Baseline - Supine
F3	Transition – supine to standing
F4	Start of Standing
F5	End of Standing – 1 min
F6	End of Standing – 3 min
F7	End of Stimuli
F8	Start of Baseline 1 - Handgrip
F9	End of Baseline 1 - Handgrip
F10	Start of Baseline 2 - Handgrip
F11	End of Baseline 2 - Handgrip
F12	Start of Baseline 3 – Handgrip
Shift+A	End of Baseline 3 - Handgrip
Shift+B	MVC Session 1 Start
Shift+C	MVC Session 1 End
Shift+D	MVC Session 2 Start
Shift+E	MVC Session 2 End
Shift+F	MVC Session 3 Start
Shift+G	MVC Session 3 End
Shift+H	30% Start
Shift+I	30% End
Shift+J	50% Start
Shift+K	50% End
Shift+L	70% Start
Shift+M	70% End
Shift+N	100% Start
Shift+O	100% End
Shfit+P	Start of Baseline – Cold Exposure
Shift+Q	End of Baseline – Cold Exposure
Shift+R	Start of Cold Exposure
Shift+S	End of Cold Exposure
Shift+T	Start of 5 min post-cold exposure
Shift+U	End of 5 min post-cold exposure
Shift+V	Start of 10 min post-cold exposure
Shift+W	End of 10 min post-cold exposure
Shift+X	Void data

END OF DOCUMENT
