# Supplementary material for: Magnitude and variability of blood pressure and renal vascular conductance responses to postural changes, exercise, and cold in black adults: A pilot study
Source: Physiol Rep. 2026 May 5;14(9):e70888. doi: 10.14814/phy2.70888 (PMC13144747; doi:10.14814/phy2.70888)
Supplement: Supplementary file 4 — Supplemental Doc 2. [file PHY2-14-e70888-s002.pdf]

DOCUMENT IDENTIFIER<br>**PROTOCOL 19-I-0093 Renal Doppler SOP**	ISSUED BY<br>Department of Radiology and Imaging Sciences
EFFECTIVE DATE<br>09/16/2025	FREQUENCY OF REVIEW<br>As Needed

**Revision History**		
**Version No.**	**Effective Date**	**Description**
**2**	September 16th, 2025	**Minor edits to instructional language, addition of team member**
**1**	November 11th, 2023	**SOP detailing procedures for renal doppler ultrasound in the NIH Clinical Center**

Written by Brittany Harrison, RDCS, RVT, RDMS, CIIP (Lead Ultrasound Technologist), Mohamed Ibrahim, (IRTA), Parker Ruhl, MD (Staff Clinician), and reviewed by Hans Ackerman MD, DPhil, MSc (Chief, Physiology Unit).

Approved For Use For Protocol 19-I-0093 By: **Date:**

AMY P. RUHL -S Digitally signed by AMY P. RUHL -S Date: 2025.09.16 23:43:10 -04'00'

______________________________________ ____________________

A. Parker Ruhl, MD, Staff Clinician
(Protocol 19-I-0093)

Ultrasound – Renal Doppler Evaluation for PROTOCOL 19-I-0093

PURPOSE:
Sonographic evaluation of the renal vasculature to evaluate vascular physiology in otherwise healthy individuals with hemoglobin genetic variants such as alpha globin gene deletions (alpha thalassemia trait and carrier status) and sickle cell trait. The evaluation is conducted under research protocol 19-I-0093 which is designed to explore the role of endothelial hemoglobin located in human micro-vasculature and its effect on the regulation of nitric oxide (Ruhl et al., 2022).

SCOPE:
- Applies to ultrasound imaging request US RENAL VASCULAR under protocol 19-I-0093
- This protocol includes color and pulse wave doppler of the bilateral kidneys at baseline and with hand grip and cold exposure stimuli

INDICATIONS:
Examination of subjects participating in research protocol 19-I-0093

CONTRAINDICATIONS:
Failure to meet the requirements necessary for participation in protocol 19-I-0093

LIMITATIONS:
Failure to adhere to subject preparation
Inability to follow instructions pertaining to handgrip and/or cold exposure stimulus
Body habitus/obesity
Any wound or dressing obscuring or covering area of interest
Extreme pain or discomfort while imaging area of interest

EQUIPMENT:
- Transducer/probe:
  - **C1-6VN -** 1.0 MHz - 6.0 MHz –optimal penetration and resolution for evaluation of the renal vasculature
- Ultrasound Gel
- Exam table paper, disposable, or reusable sheet
- Towels
- Gloves

SUBJECT PREPARATION:
Subjects are required to be NPO for 8 hours prior to study to minimize bowel gas and abdominal distention

EXAM TECHNIQUES:
- Deep inspiration facilitates inferior displacement of the diaphragm, allowing for improved subcostal imaging of the right kidney
- Left lateral decubitus (LLD) positioning allows the right kidney to shift medially, optimizing visibility of the right lower pole
- Examine the left kidney from a posterolateral or direct lateral approach in the right lateral decubitus (RLD) position
- Cine loops, in addition to still images, should be used when evaluating critical, focal, or indeterminate findings.

- The kidneys can be evaluated from a supine or decubitus position using the liver and spleen as acoustic windows for optimal image resolution
- Color flow Doppler can be used to elicit a “twinkle artifact” in the setting of renal calculi
- Please make note of the following doppler calculation package abbreviations
  - **PSV** Peak systolic velocity
  - **EDV** End diastolic velocity
  - **AT** Acceleration time
  - **RI** Resistive index
- **RENAL VASCULATURE EVALUATION**
  - The angle corrected, aortic peak velocity must be measured proximal to the renal artery origin
  - Be sure to evaluate the aorta for multiple renal arteries
    - Accessory renal arteries constitute the most common renal vascular variant. While a single renal artery supplies the kidney in 70% of the population, variation exists in the remaining 30%.
    - Notably, multiple renal arteries are three times more likely to be a unilateral finding, rather than bilateral.
    - If multiple renal arteries are identified the contralateral renal artery must be used for the HG stimulus.
  - Adjust the color doppler scale to maximally fill the vessel without artifacts (i.e., aliasing, color blooming/bleed)
  - Use the best approach to visualize the renal arteries- this varies from subject to subject
    - Evaluate the renal arteries branching from the transverse abdominal aorta (see appendix)
    - Evaluate the renal arteries in the coronal view, with the subject in the RLD or LLD position (see appendix)
  - Optimize spectral doppler tracing to eliminate aliasing and clearly demonstrate peak and end diastolic measurements
  - Identification of color aliasing, “parvus- tardus” waveforms, and elevated peak velocities are suggestive of stenosis
    - Consider that the majority of renal artery stenoses in adults occur at the ostium
  - All velocities must be angle corrected
    - Measure all velocities with the lowest feasible angle of insonation
    - The angle correction should be in line with the flow direction, therefore, try multiple imaging approaches to optimize the angle
- **HANDGRIP (HG) STIMULUS**
  - The subject must be in a supine position
  - The renal vasculature is evaluated in a transverse plane using an anterior approach (see appendix)
  - The right renal artery is routinely used when obtaining velocities during the HG stimulus
    - In the setting of multiple renal arteries, use the contralateral renal artery
  - Minimize the amount of pressure used during renal vascular interrogation, as it may cause extrinsic pressure yielding false blood pressure readings
  - The subject will be instructed to apply pressure to the HG device at varying pressures for a duration of 15 seconds. A continuous doppler sample will be obtained for the entire duration of the HG
    - The research team will provide a verbal “start” cue to begin sampling after which the doppler will commence
    - The research team will provide a verbal “image” cue to end sampling after which the image will be frozen and measured (PSV, EDV, RI, AT)
    - These steps will be repeated for Sample 1 – PROX MRA, and Sample 2- DIST MRA during BASELINE HG, MAX HG, 30% HG, 50% HG, 70% HG and 100% HG

      ***NOTE**: Baseline HG – 3 samples obtained / Max HG – 3 samples obtained
    - Using the trackball to scroll through the sample, measure the PSV, EDV, AT, and RI at the middle of the sample – between seconds 5-10

        - Be sure to choose the waveform that most clearly discerns PS and ED flow
- **COLD EXPOSURE STIMULUS**
    - The subject must be in a supine position
    - Baseline measurement will be taken over a 15 second window while the subject lie supine with their hand flat by their side, not submerged in water
        - The research team will provide a verbal “start” cue to begin sampling after which the doppler will commence
        - The research team will provide a verbal “image” cue to end sampling after which the image will be frozen and measured (PSV, EDV, RI, AT)
    - While remaining in the supine position, the subject will be instructed to submerge their hand in water for 60-seconds
        - At 45 seconds
            - The research team will provide a verbal “start” cue to begin sampling after which the doppler will commence
            - The research team will provide a verbal “image” cue to end sampling after which the image will be frozen and measured (PSV, EDV, RI, AT)
    - The research team will instruct the subject to remove their hand from the cold stimulus
        - At 5 minutes post submersion
            - The research team will provide a verbal “start” cue to begin sampling after which the doppler will commence
            - The research team will provide a verbal “image” cue to end sampling after which the image will be frozen and measured (PSV, EDV, RI, AT)
        - At 10 minutes post submersion
            - The research team will provide a verbal “start” cue to begin sampling after which the doppler will commence
            - The research team will provide a verbal “image” cue to end sampling after which the image will be frozen and measured (PSV, EDV, RI, AT)
    - It is imperative to document any notable changes in renal artery velocity over this time
    - Using the trackball to scroll through the sample, measure the PSV, EDV, AT, and RI at the middle of the sample – between seconds 5-10
        - Be sure to choose the waveform that most clearly discerns PS and ED flow

<u>EXAM INITIATION</u>:

- Begin exam in the CRIS system
- Select the subject from the worklist generated on the ultrasound machine
- Make necessary exam supplies readily available (i.e., ultrasound gel, gloves, sheets, etc.)
- Verify subject identity using their wristband or verbal confirmation of their name and date of birth
- Introduce yourself to the subject
- Be prepared to answer any questions from the subject or research team

<u>EXAM DOCUMENTATION</u>:

Aorta

- Long gray scale
- Long color doppler
- Long color doppler, spectral doppler, measure PSV

Right Kidney

- Longitudinal image of the right liver/kidney interface
- Longitudinal image of the right kidney midline with length measurement
- Longitudinal Color doppler image of right kidney midline
- Longitudinal images of the right kidney medial and lateral

- Transverse images of the right kidney upper, mid, and lower pole
- **Right Renal Vasculature**
  - Main renal artery
    - Proximal, mid, distal grayscale
    - Proximal, mid, distal color
    - Proximal, mid, distal color doppler, spectral doppler, measure PSV, EDV, AT, RI
  - Main renal vein
    - Gray scale
    - Color doppler
    - Color doppler, spectral doppler
  - Interlobar artery
    - Upper pole, mid and lower pole color doppler, spectral doppler, measure PSV, EDV, AT, RI
  - Accessory renal artery (if visualized)
    - Proximal, mid, distal grayscale
    - Proximal, mid, distal color
    - Proximal, mid, distal color, spectral doppler, measure PSV, EDV, AT, RI

Left Kidney

- Longitudinal image of the left kidney midline with length measurement
- Longitudinal Color doppler image of left kidney midline
- Longitudinal images of the left kidney medial and lateral
- Transverse images of the left kidney upper, mid, and lower pole
- **Left Renal Vasculature**
  - Main renal artery
    - Proximal grayscale
    - Proximal color
    - Proximal color, spectral doppler, measure PSV, EDV, AT, RI
  - Main renal vein
    - Color doppler, spectral doppler

Handgrip (HG) Stimulus

- **Proximal Right Renal Artery**
  - Color Doppler, spectral doppler, measure PSV, EDV, AT, RI
    - Baseline HG (x3)
    - MRA MAX HG (x3)
    - MRA 30% HG
    - MRA 50% HG
    - MRA 70% HG
    - MRA 100% HG

Cold Exposure Stimulus

- Proximal Right Renal Artery
  - Color Doppler, spectral doppler, measure PSV, EDV, AT, RI
    - At baseline (just before cold exposure)
    - During submersion - at 45-seconds
    - 5 minutes after hand has been removed
    - 10 minutes after hand has been removed

POST EXAM:

- End exam on ultrasound machine- this action will transfer all images to PACS
- Provide subject with towels to wipe any excess gel – help when needed
- Review exam images and complete corresponding worksheet
- Scan worksheet and end study in the CRIS system

- Present subject history and exam details to the reading radiologist
- Thank the subject for their patience and cooperation
- Clean ultrasound equipment and exam tables with designated disinfecting wipes

References

Granata, A., Fiorini, F., Andrulli, S., Logias, F., Gallieni, M., Romano, G., Sicurezza, E., & Fiore, C. E. (2009). Doppler ultrasound and renal artery stenosis: An overview. *Journal of Ultrasound*, *12*(4), 133–143. https://doi.org/10.1016/j.jus.2009.09.006

Kliewer, M. A., Tupler, R. H., Hertzberg, B. S., Paine, S. S., DeLong, D. M., Svetkey, L. P., & Carroll, B. A. (1994). Doppler evaluation of renal artery stenosis: Interobserver agreement in the interpretation of waveform morphology. *American Journal of Roentgenology*, *162*(6), 1371–1376. https://doi.org/10.2214/ajr.162.6.8192002

R Ruhl, A. P., Jeffries, N., Yang, Y., Naik, R. P., Patki, A., Pecker, L. H., Mott, B. T., Zakai, N. A., Winkler, C. A., Kopp, J. B., Lange, L. A., Irvin, M. R., Gutierrez, O. M., Cushman, M., & Ackerman, H. C. (2022). Alpha globin gene copy number is associated with prevalent chronic kidney disease and incident end-stage kidney disease among Black Americans. *Journal of the American Society of Nephrology*, *33*(1), 213–224. https://doi.org/10.1681/asn.2021050653

APPENDIX:

Transverse, anterior approach

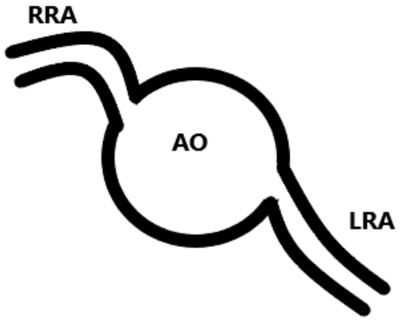

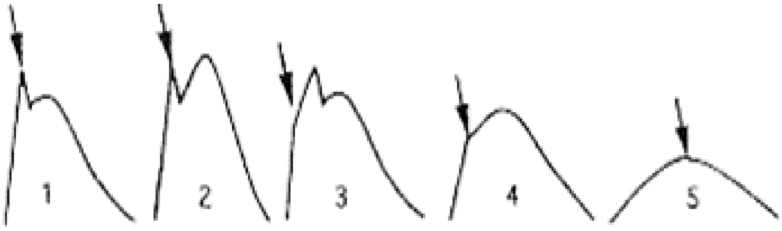

Measure acceleration in early systole; if break in systolic upstroke, measure at break (#3 and 4); if no break; measure at highest first peak (#1,2,5)

Kliewer et al., 1994

Renal artery diameter reduction	Renal artery PSV	RAR
Normal[a]	<180 cm/s	<3.5
<60%	>180 cm/s	<3.5
≥60%	>180 cm/s	≥3.5
Occlusion	No signal	Indeterminable

[a] PSV = 100 ± 20 cm/s.

Granata et al., 2009

GRADING CRITERIA	
**PSV**	<180 cm/s
**RENAL AORTIC RATIO (RAR)**	< 3.5
**RESISTIVE INDEX (RI)**	<0.70
**RI (right – left)**	<0.05
**ACCELERATION TIME (AT)**	<0.07
**ACCELERATION INDEX (AI)**	>3.5m/s²
